# Stated benefits of teleworking in Mexico City: a discrete choice experiment on office workers

**DOI:** 10.1007/s11116-022-10293-w

**Published:** 2022-05-08

**Authors:** Jose Alberto Lara-Pulido, Adan L. Martinez-Cruz

**Affiliations:** 1 Centro Transdisciplinar Universitario para la Sustentabilidad (CENTRUS), Universidad Iberoamericana, Mexico City, Mexico; 2 Department of Forest Economics and Centre for Environmental and Resource Economics (CERE), Swedish University of Agricultural Sciences (SLU), Umeå, Sweden; 3 Department of Economics, Centro de Investigacion y Docencia Economicas (CIDE), Mexico City, Mexico

**Keywords:** Remote working, Shared office, Value of commuting time, Value of bike parking infrastructure, Megacities, Mexico City, R00, R39, R41

## Abstract

Commuting is expensive in megacities of emerging economies. By decreasing work-related trips, teleworking may reduce congestion and commuting time. Taking Mexico City’s office workers’ as case study, this paper reports findings from a discrete choice experiment (DCE) exploring willingness to see a cut in monthly paycheck in exchange for teleworking two days a week from a shared office. This DCE explores preferences for bike parking spaces at shared office’s facilities, and walking commuting time to shared office. This design allows estimation of willingness to pay (WTP) for teleworking across commuting time scenarios. Monthly WTP for teleworking 2 days a week starts at (2019) USD 76.68—if commuting time is zero. As 1 h of commuting time is valued at USD 61.97 on a monthly basis, WTP for teleworking 30 min away from home is USD 45.69. Wealthier respondents report higher value for commuting time and WTP for teleworking. Monthly value of bike parking infrastructure is USD 14.70—reaching USD 30.98 for commuters that walk or (motor-)bike less than 50 min. We illustrate how these stated benefits can inform cost-benefit analysis of transportation, housing, and labor policies that enable teleworking and/or reduce commuting times in Mexico City.

## Introduction

Commuting is expensive in emerging economies’ megacities such as Mexico City, New Delhi, Sao Paulo, Manila, Nairobi, and Accra (Hess and Narteh-Yoe 2020). In Mexico City (CDMX), for instance, transportation expenses can consume up to a third of a salary of a minimum-wage worker (Guerra 2017), and as much as 40% of an annual middle-income class salary (Arredondo 2017). The majority of work-related trips cover at least 15 kms (Graizbord 2015). Time-wise, commuting by car in 2019 wasted 195 h when driving in rush hours (TomTom 2020).

Teleworking (or remote work) may reduce average commuting times by decreasing work-related trips and, in turn, alleviating congestion during peak hours (Zhang et al. 2020). Teleworking refers to work arrangements under which an employee performs duties and responsibilities of his/her position, and other authorized activities, at an approved (by his/her employer) location other than his/her employer’s premises (USOPM 2020). Teleworking arrangements most commonly have involved working from home, but emergence of mobile devices such as laptops and mobile phones has enabled remote work from practically any location (López-Igual and Rodríguez-Modroño 2020).

A question that naturally arises in this context is whether office workers in megacities of emerging economies would be willing to see a cut in their monthly paycheck in exchange to work remotely. To the best of our knowledge, no previous study has paid attention to this issue. Taking CDMX’s office workers as a case study, this paper explores stated willingness to pay for teleworking—a discrete choice experiment (DCE) has been implemented on a sample of 1,179 office workers from November to December 2019. Office workers in CDMX represent an illustrative population for purposes of our research question because around 80% of commuters in CDMX would be willing to make changes in their daily life so that commuting time is reduced, and 88% are aware of negative impacts that long-time commuting has on their quality of life and health (Hess and Narteh-Yoe 2020). Also, around 38% of office workers in CDMX can potentially perform their job remotely (Monroy-Gómez-Franco 2020).

Our DCE has described a scenario in which respondent’s employer offers him/her the opportunity of teleworking two days a week from a shared office in exchange for a cut in monthly paycheck. This focus in shared offices as the premises to work remotely is in itself a contribution to the literature that has studied teleworking. This longstanding literature has almost exclusively focused on home as the premise where office workers can work remotely (see Aguilera et al. 2016; Bojovic et al. 2020; Chang 2019; López-Igual and Rodríguez-Modroño 2020; Zhang et al. 2020). Exploring alternative premises to home-based teleworking is worth the effort because teleworking from home has been associated with risks such as emotional isolation, mental and physical conditions, and loss in productivity—originated in a lack of a healthy division between work and personal domains (ILO 2020). By providing an out-of-home space that allows a clear division between personal and work domains, a shared office may contribute to reaching a healthy work-life balance. The risk of emotional isolation and associated mental health concerns are likely lower if a person works in a shared office, where other people are carrying similar tasks and social interaction is feasible. If a shared office is located within a reasonable commuting time from home—where *reasonable* depends on commute mode alternatives and workers’ preferences—, physical activity may also increase in comparison to working from home and at employee’s premises—for instance, a close-to-home shared office may incentivize workers to commute by bike or e-bike, or walking.

Our focus on shared offices is also motivated by living conditions in megacities of emerging economies. For instance, overcrowded dwellings in CDMX make office-related activities difficult to perform—some of these activities may require privacy that overcrowded dwellings cannot provide. In addition, quality of internet connection varies widely across CDMX’s municipalities. In this context, a shared office provides office conditions (e.g. office equipment, internet connection) that a person living in an overcrowded dwelling can only provide his/herself at large individual costs.

Overcrowded homes and low quality internet are two features that hold across megacities in emerging economics, and thus our focus on shared offices as premises to work remotely is of interest in other megacities. That is, estimates reported in this paper can inform cost-benefit analysis of measures enabling teleworking and/or decreasing commuting times not only in CDMX but they may also be used to inform benefit transfer exercises.

## Related literature

This section places the contribution of this study within three literatures. One refers to the literature that has documented barriers to and potential benefits from teleworking. This strand in the literature has mostly focused on home-based teleworking, overlooking potential benefits from (and barriers to) remote work from other locations.

The second literature of interest infers value of commuting time based on stated preferences with respect to transportation scenarios. This paper’s contribution to this strand of the literature is twofold. On one hand, it contributes by documenting the value of commuting time in a megacity located in an emerging economy—previous studies have mostly focused on cities located in developed countries. As a second contribution to the transportation literature, this paper belongs to a small number of discrete choice experiment studies taking advantage of the trade-off between commuting time and wage to infer value of commuting time—studies within this literature most frequently analyze between-mode choices or within-mode choices.

The third literature of interest in this section refers to the literature documenting determinants of commuting time/distance. The link of this paper to such literature arises from the fact that preferences for teleworking alternatives and preferences for commuting time/distance are likely shared with opposite signs—i.e. factors positively associated with commuting time/distance are likely expectedly negatively associated with preference for teleworking.

### Teleworking

For purposes of this paper, two strands of teleworking literature are of interest. As teleworking has been posed as an alternative to decrease commuting trips and consequently congestion, the first strand of teleworking literature that we cover here is the one discussing and documenting effects of teleworking on congestion and energy consumption. The second strand refers to studies documenting barriers to a widespread adoption of home-based teleworking—which is relevant to this study because we suggest that shared offices may represent an option to overcome barriers faced by home-based teleworking.

#### Energy savings associated with teleworking

For nearly five decades, teleworking has been promoted as one of the traffic demand management policies that can potentially alleviate congestion during peak periods and reduce work-related trips, along with other benefits (Hook et al. 2020; Zhang et al. 2020). Indeed, while work-related trips unambiguously decrease when teleworking is in place, there is the possibility that non-work related trips increase—with implications on to what extent congestion can be reduced—and/or energy use associated with home-based teleworking is higher than energy use associated with regular commuting patterns (Hook et al. 2020).

This issue has been under recent scrutiny through two systematic reviews—carried out separately, and with an exclusive focus on home-based teleworking. Hook et al. (2020) explore whether teleworking reduces work-related trips and consequent impacts on economy-wide energy consumption. They review 39 studies documenting energy savings from reduction in work-related trips and indirect impacts on energy consumption associated with changes in non-work travel and home energy consumption. According to Hook et al. (2020), 26 studies document a reduction in energy use associated with home-based teleworking, and 8 studies suggest an increase in energy use or a neutral impact. Importantly for purposes of this study, the main source of energy savings identified by this literature is the reduced distance in commuting trips—with a modest contribution from reduction in energy use at office premises. When zooming in studies that include wider range of impacts such as non-work travel and home energy use, Hook et al. (2020) document smaller energy savings in general.

O’Brien and Aliabadi (2020) explores whether teleworking promotes energy savings and reduction of greenhouse gases. They do so through a literature review that accounts for energy use associated with home-based teleworking, internet use, long-term consumer choices, and a rebound effect in several domains. O’Brien and Aliabadi (2020)’s conclusion points to a limitation in current datasets and methods to adequately address the issue at a economy-wide scale. From O’Brien and Aliabadi (2020)’s perspective, most studies indicate net benefits to some extent, but there are enough studies indicating an increase in energy use in several sectors, including transportation—which is the domain in which most benefits would have been expected.

In general, available evidence suggest that economy-wide energy savings from home-based teleworking are positive but modest. Importantly, Hook et al. (2020) and O’Brien and Aliabadi (2020) warn that, due to several uncertainties, there is a probability that energy savings could be negative. While the main source of savings is reduced distance travelled for commuting purposes and lower office energy consumption, the potential for negative energy savings arise from the possibility that teleworking increases home energy use and non-work travel made by members of teleworkers’ household.

#### Obstacles to home-based teleworking

Teleworking has long been expected to become a popular working arrangement. But it has not occurred so far. Before COVID-19 hit, only 9% of USA’s working population teleworked once a week (Zhu et al. 2018); in Europe, this number was around 5% in 2017 (López-Igual and Rodríguez-Modroño 2020)—with national numbers reaching up to 30% in Denmark, Netherlands, and Sweden (ILO 2020)—; 16% in Japan, and only 1.6% in Argentina (ILO 2020).

From employees’ point of view, barriers to adoption of teleworking include concerns about work-life balance, emotional isolation (particularly in the case of individuals living alone), and fearing that teleworking may imply missing out opportunities for career advancements (Golden et al. 2008; Schulte 2015). These concerns are not unwarranted. For instance, the impact on work-life balance may go both ways because teleworking blurs boundaries between paid work and personal life domains (ILO 2020). Also, employers tend to increase work load of teleworkers (Noonan and Glass 2012; Russell et al. 2009). In addition, teleworking from home has been documented to be strongly associated with the presence of children at home, with an spillover effect aggravating gender differences in the division of housework (Thulin et al. 2019; Zhang et al. 2020)—an effect that COVID-19 has brought to the forefront (see ILO 2020).

From employers’ point of view, teleworking represents challenges to accountability and measurement of productivity (Pérez et al. 2005). These challenges translate into an increase in managers’ responsibilities and the corresponding time handling them. For instance, Microsoft in China has calculated that leading teleworking teams adds an extra 90 min per week to working time of managers—time that results from additional one-to-one calls and meetings (Sapataro 2020). COVID-19 has made clear that an important barrier at the firm level is the lack of appropriate IT tools and devices, and lack of skill and training resources (ILO 2020).

Additional barriers stem from concerns about data security and privacy issues (see ILO 2020)—a particularly difficult obstacle in emerging economies with unclear regulatory frameworks, and weak laws protecting intellectual property rights and confidentiality of sensitive data (Mitter 2000).

This paper puts forward that teleworking from a shared office may represent an alternative to overcome some of the challenges faced by home-based teleworking. For instance, people that live alone would likely face less risk of emotional isolation if they work from an shared office, where other people perform similar tasks. Also, work-life balance may be easier to reach if a person works in an out-of-home space that allows a clear division between personal and work domains. A healthy work-life balance would be more reachable if the office space is within reasonable commuting time as it would imply savings in commuting time that a worker can allocate to activities that improve his/her individual health, and/or social life. Depending on available infrastructure, less commuting time may incentivize workers to commute by walking, biking, or e-biking—with the corresponding effects on individual health and social welfare. Importantly in the context of megacities in emerging economies, the office conditions that a shared office space provides can hardly be provided by the employee him/herself if living in an overcrowded dwelling.

The scenario that this paper presents to respondents implies that teleworking would be allowed two days a week. We have included this feature to address the challenge that employees see in home-based teleworking in terms of missing out career opportunities. This hybrid arrangement leaves space for workers to interact with their peers and managers on regular basis, and in this way keep an eye to identify opportunities for promotion.

From an employers’ point of view, a shared office offers the possibility of taking advantage of economies of scale to afford associated costs. This possibility holds either if we think that several employers may share costs of renting a shared office building; or if we think that private or public companies provide shared office services, and employers pay a membership for each employee using the services.

### Inferring value of commuting time from commuting time-wage trade-off

The interest on the value of time is longstanding—with Becker (1965), Beesley (1965), and DeSerpa (1971) credited as pioneers of the microeconomic modelling of time allocation (Dubernet and Axhausen 2020). Current practice in the transportation literature heavily relies on stated preferences gathered through discrete choice experiments (DCE) (Beck et al. 2017; Dubernet et al. 2020). Supported by the random utility framework, researchers most frequently take advantage of single-journey, short-term decisions to estimate the value of commuting time—either between-mode choices^1^ or within-mode choices^2^ (Beck et al. 2017; Swärdh and Algers 2016).

In the transportation literature, there are few DCE studies that have inferred the value of commuting time by analyzing the trade-off between commuting time and wage (Swärdh and Algers 2016).^3^ This trade-off has presented itself as a natural candidate in our application because, as a by-product of our main objective, we explore how much wage office workers would give up under a range of commuting times—i.e. what is the commuting time that office workers would find reasonable if a shared office that is *near* their home is offered to them.

In this respect, our DCE is closest to the one designed by Swärdh and Algers (2016). In 2005, they presented both earners in a sample of two-earner households residing in the Stockholm region, Sweden, to scenarios in which respondents receive offers trading-off longer commuting time and a higher wage—i.e. respondents chose to accept or to reject an offer where both wage and commuting time increase. Their main contribution arises from treating respondents with two different stated choice experiments. In the first one, respondents report their willingness to accept longer commuting time themselves—i.e. as if the offer was made to the respondent. In the second choice experiment, respondents report their willingness to accept or to reject offers where both the respondent and his/her spouse simultaneously receive wage increases as compensation for longer commuting. With this split-sample approach, they aim to identify gender differences in respondent’s value of his/her own commuting time compared to the respondents’ value of his/her spouse’s commuting time. They document that when respondent’s own commuting time and attributes are the DCE’s attributes, the estimated value of commuting time does not differ between men and women. In contrast, when valuing spouses’ commuting time, both spouses value the commuting time of the wife highest.

Beck et al. (2017) and Dubernet et al. (2020) have pointed out that a decision between commuting distance/time and wage is a decision with lasting implications in terms of travel patterns of the decision makers because it changes choice sets for future short-term decisions over periods that typically cover several years or even decades. Thus, Beck et al. (2017) have borrowed the data gathered by Swärdh and Algers (2016) to document significantly higher values of commuting time when scenarios involve long-term decisions in comparison to short-term ones. Pursuing a similar goal than Beck et al. (2017), Dubernet et al. (2020) analyze stated decisions to scenarios presented by the German Value of Time and Reliability Survey because this survey not only have presented salary gains—as in Swärdh and Algers (2016)—but salary losses as well as salary neutral scenarios. They document opposite effects to those reported by Beck et al. (2017) when salary neutral situations are considered.^4^

In general, the literature on value of travel time has focused on developed economies—e.g. a recent literature review focused only on European cases has been able to cover 3,109 estimates reported in 389 studies (Wardman et al. 2016). This focus is not different when zooming on the few studies using DCE to infer value of commuting time from the wage-commuting time trade-off.

### Factors associated with commuting time/distance

In this paper, we explore preferences for teleworking and value of commuting time. Factors associated with these preferences expectedly intersect with those factors associated with revealed commuting time/distance. Thus, this section briefly describes the main factors identified by the literature on commuting time/distance.

Gender, education, wealth-related variables, marriage, age, presence of other household members, and interactions among these factors have long been identified as associated with commuting time and distance. Lee and McDonald (2003), for instance, document on a 1995’s sample of Seoul residents that commuting distances and times are longer for male commuters, full-time salaried workers, commuters with more education, home-owners, and male workers older than 35 years old. Lee and McDonald (2003) also document that shorter commuting times and distances are observed for married women, self-employed, and part-time workers. Also, gender differences in commuting time are documented to be wider among married workers with less education, and the presence of parents-in-law in the household is associated with longer commuting trips by married women. Factors and patterns documented for Seoul residents are similar to those documented in Los Angeles’ residents during the 90’s (see Giuliano 1998).

Similar findings in other contexts have followed suit. For instance, Zhao and Cao (2020) have analyzed 81 million trips from 28 million transit smart cars in 2015’s Shanghai. In terms of wealth-related factors, via a geographically weighted regression, they document that workers with long commutes tend to live in disadvantaged areas characterized by low rent or poor job accessibility. Indeed, this commuters face a trade-off between housing prices and travel costs.

Intersections between factors discussed above and race and/or migration condition have also long been documented. Focusing in New York commuters in 1980 and 1990, McLafferty (1997) documents that presence of children at household leads to shorter trips for white women in the suburbs of New York; and for men of all race/ethnic groups, marriage lengthens commuting times. Zhao and Cao (2020) documents that workers with shorter commutes live in areas with larger migrant populations in 2015’s Shanghai.

Using 2017 data, Hu (2021) has investigated interactions between household types and race/ethnicity on gender differences in commuting distances and probability of private car commute in United States. Five household types are considered: one adult without children, two-adult one-worker without children, two-adult two-worker without children, two-adult one-worker with children, and two-adult two-worker with children. Hu (2021) documents that both gender gap and variation in this gap associated with household type tend to be the smallest for Black households; and gender gap varies the greatest with household type for Hispanic households. The author interprets these results as arising from the role that families play in the Hispanic community in terms of enabling women’s commuting behavior to a greater extent than women in other race/ethnicity group. For the case of Black households, their relatively egalitarian gap is interpreted as arising from economic and spatial depressions for both men and women. Specific findings include that the only group with longer commute distance for women than men are Black workers in two-worker households without children. In other types of Black households, gender gaps in commuting distance exist only in two-worker households with children, and the gap is relatively small.

Focusing on two-worker households in the San Francisco Bay Metropolitan Area, Sermons and Koppelman (2001) explore the interaction between residential location decisions and commuting times. They do so by developing a multinomial logit that models residential location choices as a function of commuting time, and commuting time, in turn, as a function of household characteristics. They document that presence of children, occupation of the male worker, and the relative order of the last residential change and the last change in the female worker’s workplace are determinants of female and male commuting times parameters in household residential location utility functions.

## Methodological approach

Our empirical approach relies on the Random Utility Model (RUM) as it provides theoretical support to empirical analysis of discrete choice experiments (Train 2009). In our context, office workers are presented to the task of choosing from among three teleworking alternatives, one of which is no teleworking at all—and instead commuting to his/her employer’s facilities as usual. The RUM departs from the assumption that, when inquired to choose from among mutually exclusive alternatives, an office worker chooses the alternative that provides him/her with the highest utility. Formally, assume that worker *i*’s indirect utility from alternative *j* is denoted as $U_{ij}$, for $i = 1,2,\ldots ,I$ and $j = 1,2,\ldots ,J$. The utility provided by the chosen alternative is the maximum from among available alternatives, i.e.

1 $$\begin{aligned} U_i^{max} = max\{U_{i1}, U_{i2},\ldots , U_{iJ}\} \end{aligned}$$

An additional assumption is that the utility that an office worker obtains from a teleworking alternative results from adding (marginal) utilities that each component (or attribute) of the specific alternative provides. In our context, for instance, an office worker may obtain utility from not working at his/her employer’s facilities (attribute one), from working closer to home (attribute two), and from having access to infrastructure to park bikes at a teleworking facility (attribute three); and an office worker most likely experiences disutility from having to pay for the opportunity to telework (attribute four). Assuming that a researcher is able to infer utilities and/or dis-utilities from each of these four attributes, then the utility that a teleworking alternative provides can be calculated by adding these (marginal) utilities. Formally, this explanation translates into the assumption that worker *i*’s indirect utility can be represented as a linear function. That is $U_{ij} = {\beta }^{'} x_{ij}$, where $x_{ij}$ is a $M \times 1$ column vector denoting *M* alternative-specific attributes—one of which can be an alternative-specific intercept representing, for instance, a generic alternative of not working at employer’s facilities—; and $\beta$ is a $M \times 1$ column vector representing preferences for alternative-specific attributes.

Office workers are assumed to know their own utility function with certainty—i.e. they know exactly why they prefer a given alternative over the rest. Researchers, however, cannot fully observe each $U_{ij}$. Thus a random component needs to be incorporated into this utility model. Formally, $U_{ij}$ is approached by researchers as a random linear function:

2 $$\begin{aligned} U_{ij} = V_{ij} + \epsilon _{ij} = {\beta }^{'} x_{ij} + \epsilon _{ij} \end{aligned}$$

where $V_{ij}={\beta }^{'} x_{ij}$ is the component observed by researchers; and $\epsilon _{ij}$ represents the purely random heterogeneity that the researcher is unable to observe.

Under the assumptions embedded in Eq. (2), a researcher cannot observe $U_{i}^{max}$ as defined in Eq. (1). A researcher can only make statements in terms of expected utilities which are calculated over the error term $\epsilon _{ij}$, i.e.

3 $$\begin{aligned} E(U_i^{max}) = E_{\epsilon }[max\{V_{i1}, V_{i2},\ldots , V_{iJ}\}] \end{aligned}$$

Under the assumption that $\epsilon _{ij}$ follow a type I extreme value distribution, the expected maximum utility can be calculated through the log sum formula,^5^ i.e.

$$\begin{aligned} E(U_i^{max}) = ln{\sum _{j = 1}^{J} exp(V_{ij})} \end{aligned}$$

Accordingly, statements involving welfare measures are made in expected terms. For a before (*b*) and an after (*a*) situations—where after implies a change in the available alternatives—, the expected value of the compensation variation (CV) due to the change in worker *i*’s utility is expressed as

4 $$\begin{aligned} E_{\epsilon }(CV_i)&= \frac{1}{-\beta _p} (E_{\epsilon }(U_i^{max,a}) -E_{\epsilon }(U_i^{max,b})) \nonumber \\&= \frac{1}{-\beta _p} \left( ln{\sum _{j = 1}^{J} exp(V_{ij}^a)} -ln{\sum _{j = 1}^{J} exp(V_{ij}^b)}\right) \end{aligned}$$

where $\beta _p$ represents, in our case, the marginal (dis)utility from having to pay for the option to telework. The marginal willingness to pay (MWTP) can be derived from Eq. (4) as follows. Assume attribute *q* changes in a non-marginal fashion across all alternatives -i.e. $q^a = q^b + \Delta q$ is the level of *q* after $\Delta q$ has been added to $q^b$. This change in *q* can be thought as representing a change from status quo conditions—e.g. not teleworking alternative at all—to a generic teleworking alternative. Introduce the change in *q* in Eq. (4) and, because such a change occurs across all alternatives, factor it.^6^ The expected CV can be expressed as follows

5 $$\begin{aligned} E_{\epsilon }(CV_i[\Delta q]) = -\Delta q \frac{\beta _q}{\beta _p} \end{aligned}$$

where $\beta _q$ is the marginal utility from *q*.

Equation (5) reduces to the WTP for a marginal change across alternatives when $\Delta q = 1$—i.e. when the change in *q* is marginal, and

6 $$\begin{aligned} E_{\epsilon }(MWTP_i) = - \frac{\beta _q}{\beta _p} \end{aligned}$$

Following our illustration based in our application, Eq. (6) can be interpreted as the ratio of the marginal utility from having the option of teleworking and the negative of the marginal (dis)utility from paying to have the option of teleworking.

Empirical estimations of the parameters required in the calculation of the expected MWTP (i.e. $\hat{\beta }_q$ and $\hat{\beta }_p$) can be obtained via a conditional logit econometric specification. The focus of this empirical strategy is on estimating the probability of choosing an alternative as a function of the levels of each attribute of a teleworking alternative—in this way, a researcher can infer the relative marginal utility associated to each attribute.

Formally, the departure point of a conditional logit is the theoretical expectation of welfare measures under discrete choice modelling—i.e. $\epsilon _{ij}$ is distributed according to a type I extreme value distribution. Under this assumption, the probability that individual *i* chooses alternative *j* is expressed as follows

7 $$\begin{aligned} P_{ij}&= Pr[V_{ij} + \epsilon _{ij}> V_{ik} + \epsilon _{ik} \forall k \ne j]\nonumber \\&= Pr[\epsilon _{ij} > V_{ik} - V_{ij} +\epsilon _{ik} \forall k \ne j]\nonumber \\&= \frac{e^{V_{ij}}}{\sum _{k \in J} e^{V_{ik}}} =\frac{e^{\beta ^{'}x_{ij}}}{\sum _{k \in J} e^{\beta ^{'}x_{ik}}} \end{aligned}$$

A conditional logit (CL) specification faces two limitations to model empirical discrete choice data (Train 2009). First, a CL can represent systematic variation (i.e. taste variation that is related to observed characteristics) but not random taste variation (i.e. differences in tastes that cannot be linked to observed characteristics). Second, the estimation of the CL probabilities implies proportional substitution across alternatives—more flexible, more realistic patterns cannot be fitted with a CL model.^7^

The random parameters logit (RPL) results from adapting the CL model to incorporate non-systematic heterogeneity in preferences and discard the proportional substitution across alternatives. The RPL turns out to be a highly flexible model that can approximate any random utility model (McFadden and Train 2000).

The RPL probabilities are the integrals of standard logit probabilities over a density of parameters. That is, keeping in mind Eq. (7), a RPL is a model whose choice probabilities can be expressed in the following form

8 $$\begin{aligned} P_{ij} = \int \frac{e^{\beta ^{'} x_{ij}}}{\sum _{k \in J} e^{\beta ^{'} x_{ik}}} f(\beta ) d \beta \end{aligned}$$

where $f(\beta )$ is a density function. The RPL probability is a weighted average of the logit probabilities evaluated at different values of $\beta$, with the weights given by the density function $f(\beta )$. In statistical terms, the weighted average of several functions is called a mixed function. Consequently, a RPL is a mixture of the logit function evaluated at different $\beta$’s with $f(\beta )$ as the mixing function.

## Context, survey methods and data

### Commuting in Mexico City

Mexico City (CDMX) is the core of the Mexico City Metropolitan Area (MCMA), which is the largest metropolitan area in the Western Hemisphere and the fifth largest in the world. CDMX has been among the 10 most congested cities in the world from 2014 to 2018—in 2019, CDMX left this inglorious group to be ranked 13$^{th}$.

Based on data gathered in 2007, Guerra (2017) documents that suburban households earn 30% less than urban households, have 40% longer commutes, and spend nearly twice as much per transit trip. *Martita*’s situation is illustrative: each morning, she leaves her house on the outskirts of CDMX at 6:30 a.m. to arrive at her cleaning job by 9 a.m. This two-and-a-half hour one-way commute costs around a quarter of her daily wage (WRI 2011). Guerra (2017) documents that 80% of households use public transportation on a typical weekday and the poorest fifth of the households spend almost a quarter of their income on public transportation—twice as much as commonly used transportation affordability thresholds. Poor households are least able to reduce commuting expenditures without reducing travel because cars are expensive, non-discretionary trip distances are often too long for non-motorized modes, and wealthier households price them out of the most accessible neighborhoods.

In 2017, 34.56 million of trips were carried out in a typical week in the MCMA; 58% of them involve commuting to work place. 37% of these trips involved no more than 30 min; 58%, between 31 min and 2 h; and 5%, more than 2 h. Trips from work place to home were shorter—55% took less than 30 min; 41% took between 31 min and 2 h; and 4%, more than 2 h (INEGI 2018).

Avila-Forcada and Medina-Martinez (2019) offer a look at changes occurred to commuting patterns in CDMX during the 2007–2017 period. This decade has witnessed an increase from 14 million to 19 million in the number of MCMA’s residents, and an increase from 5.6 million to 12.27 million in the number of registered vehicles. The authors warn that, while in 2007 longer trips more likely involved public transportation, in 2017 these trips more likely involved private cars. Also, commuting by bike has more than doubled during the decade under analysis—an increase pushed by educated, older, and wealthier commuters in CDMX. They point out that, conditional on car ownership in the household, men and wealthier commuters more frequently commute by car.

Based on data collected in 2018 via online platforms, Hess and Narteh-Yoe (2020) explore the interest of commuters in New Delhi, CDMX, Sao Paulo, Manilla, Nairobi, and Acra to reduce commuting time. They report that 18% of their respondents in CDMX spend 3 h or more in commuting—a number that seems to be an overestimation in comparison to official estimates according to which 5% of commuters spend more than 2 h (INEGI 2018). Hess and Narteh-Yoe (2020) estimate the average commuting time in 2018 in CDMX at 1.8 h—with a median of 1.5 h, and a mode of 30 min. A couple of their findings work as motivation for our study: 88% of respondents in CDMX reported to be aware of negative impacts that long-time commuting has on their quality of life and health, and that 80% would be willing to make changes in their daily life so that commuting time is reduced.

We highlight that studies documenting commuting in CDMX have not explored preferences of office workers explicitly. This gap, we believe, is partially explained by the fact that the main source of information for commuting patterns in CDMX—the survey on households travel patterns—does not allow for an easy identification of office workers (see INEGI 2018).

Only until recently, and directly motivated by the challenges imposed by COVID-19 in terms of teleworking from home, Monroy-Gómez-Franco (2020) has estimated that around 38% of workers in CDMX can perform their duties remotely. Also, the author documents that salary of these workers fall in the upper tail of the hourly wage distribution—with only 5% generating a wage below the poverty line.

### Data collection

**Fig. 1:**
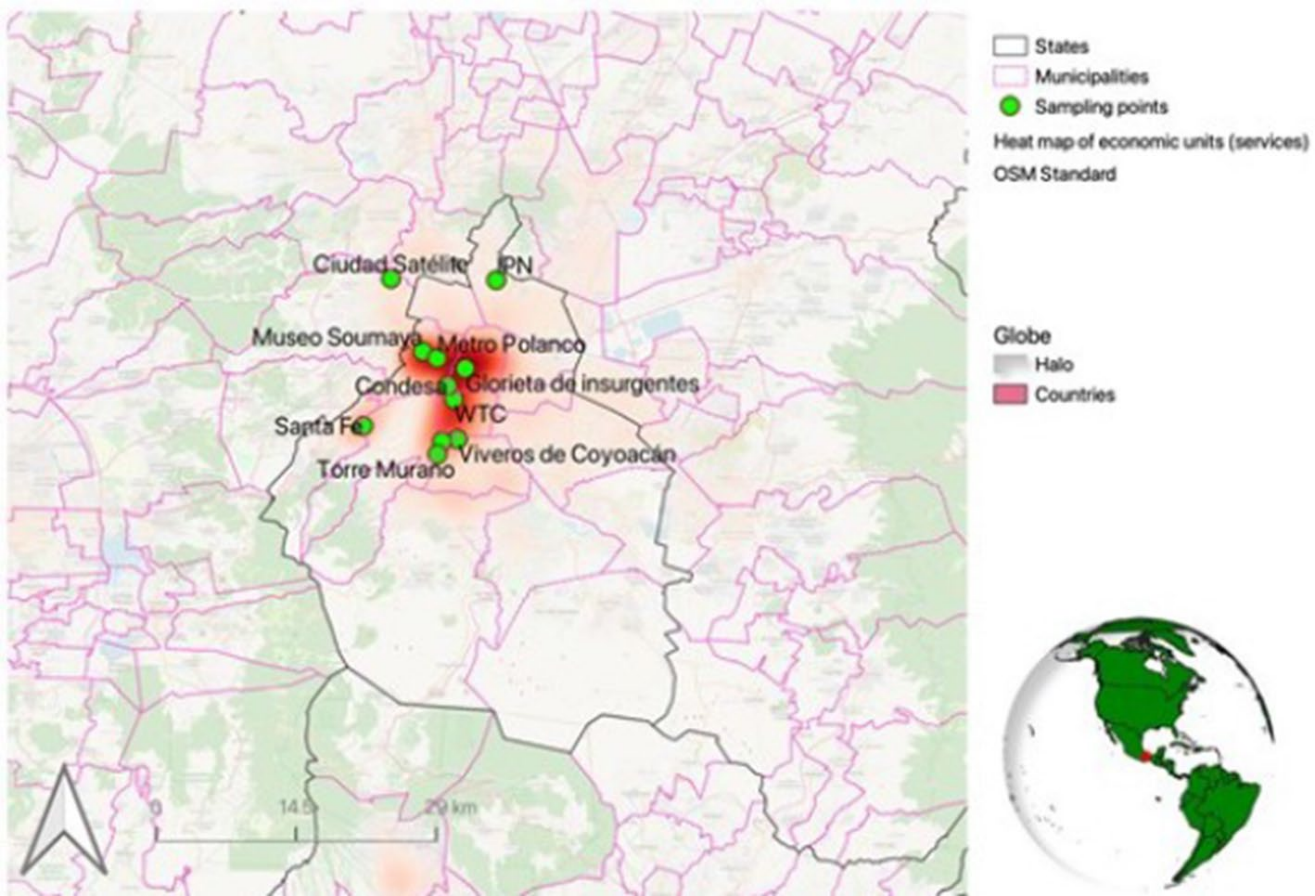
Sampling points and density of office buildings where private and public services are provided in CDMX

Face-to-face surveys were carried out from November 23 to December 19, 2019. Potential respondents were randomly approached at 11 public plazas where office workers usually hang out with colleagues to take breaks and socialize during a typical working day.^8^ These 11 spots have been selected based on the density of office buildings where both private and public services are provided in CDMX. In calculating this density, we have included the following categories of services, as reported by the Mexican Database of Economic Units (INEGI 2020): government services, communication services, corporate services, financial services, professional and scientific services. In deciding this selection criteria, we have considered both our focus on office workers and Graizbord (2015)’s insights about the sectors with the most potential to take advantage of teleworking in CDMX.^9^ Figure 1 illustrates the match between our 11 sampling points and the density of office buildings in CDMX.

The survey protocol has included one filter question so that enumerators made sure respondents were office workers in CDMX—“Do you work in an office space located in CDMX?”. The survey was composed by four sections. The first section gathered commuting information—i.e. mode(s), time, costs, and routines. The second section gathered information about respondents’ perceptions about his/her job (e.g. perception on whether he/she could perform his/her duties from a place different than employer’s premises); job-related routines (e.g. number of job-related meetings attended weekly); and type of working space (e.g. own office versus shared office space). The third section presented respondents to our discrete choice experiment. The fourth section gathered respondent’s and his/her household’s socioeconomic and demographic information.

### Discrete choice experiment

Our discrete choice experiment (DCE) initiates with a preamble that describes a shared office as follows

*The following questions gather your preferences and perceptions in case that you were offered the option of teleworking from a shared offices building which would be located near your home. These offices are also known as* coworkings^10^. *These offices are designed so that several workers can work in the same space in different moments. If interested in teleworking from a shared office, you would have access to all services, infrastructure, and comfortability needed to perform your office job.*^11^

Then, respondents have been presented to a scenario under which his/her employer offers the opportunity of teleworking two days a week from a shared office. If the respondent chooses teleworking from a shared office, then he/she would pay a monthly membership that takes the form of a monthly cut in paycheck. The respondent has been told that, whenever teleworking becomes unappealing to him/her, unenrollment is possible, and he/she can go back to work from office as usual.^12^

**Fig. 2:**
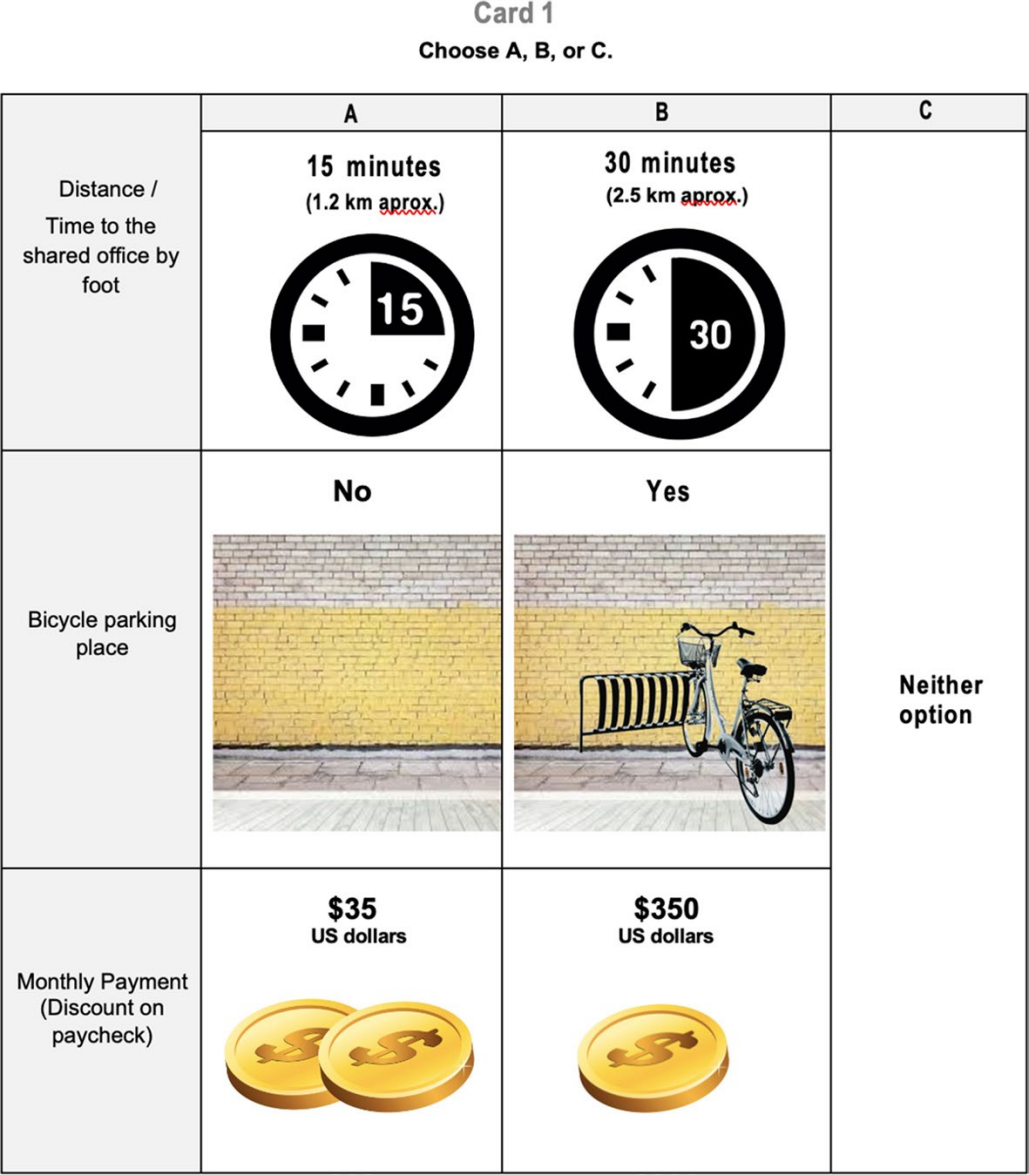
Example of a choice card. Respondents have been asked to choose their favorite teleworking alternative from among three alternatives displayed in the card, assuming that their employers have offered the option of teleworking two days a week from a shared office

Respondents have been asked to choose one of the three teleworking alternatives that are described in a choice card—Fig. 2 illustrates this choice card. Alternatives in the choice card include a status quo alternative—i.e. working from his employer’s premises. Each respondent has faced four choice cards, which belong to one of two blocks. This design has been created based on a factorial design that identifies only main effects.^13^

The two non-status quo alternatives have been described in terms of commuting time to shared office, availability of safe parking spots for bikes, and the amount that would be cut from monthly paycheck. As illustrated in Table 1, commuting time was describe as walking time and it has taken four values—15 min, 30 min, 45, min, and 60 min. Availability of parking spots for bikes is a binary attribute—taking value one when safe parking spots for bikes are available, and zero otherwise. The price attribute has taken four values—MXP 350 (USD 17.50), MXP 700 (USD 35), MXP 1,400 (USD 75), and MXP 2,500 (USD 125), on monthly basis.^14^

**Table 1:** Attributes and levels in discrete choice experiment

Attribute	Description (units)	Levels
Commuting time	Time in one-way trip by walking (minutes)	15
		30
		45
		60
Bike parking	Spots to park bikes (binary; 0/1)	No parking spots
		Parking spots
Price	Amount subtracted from monthly paycheck (MXP)	350
		700
		1,400
		2,500

We have defined final attributes of our DCE and their levels after a pilot survey on 50 respondents, and a couple of pre-pilot focus groups. In a first focus group, levels of payment and commuting distance/time were gathered through open-ended questions. In a second focus group, closed-ended questions allowed us to establish an initial range of values that seem reasonable to use in the pilot DCE. The pilot exercise has been instrumental in identifying payment values that capture upper tails of willingness to pay—in particular, our pilot DCE had MXP 2,000 as the maximum payment value but our final design extends payment up to MXP 2,500. Also, pilot implementation of our DCE has allowed us to arise to a phrasing of commuting time that respondents relate to. In particular, while we initially aimed to describe this attribute as commuting distance, pre-pilot focus groups delivered the finding that commuters in Mexico City find difficult to estimate commuting distances—partly due to congestion and non-straight commuting trajectories. Thus, we turned to commuting times, which also needed to be presented in a common unit as commuting time widely varies depending on commuting mode and other factors. Thus, our pilot played an important role in making sure that walking time is a unit that makes sense to office workers in Mexico City. We also want to mention that a pre-pilot design of our DCE included an attribute aiming to reflect features of office space itself—e.g. privacy elements and/or environmental performance. This attribute turned out to be irrelevant for office workers attending a couple of focus groups, and we dropped it in our pilot design.

### Descriptive statistics

Once observations with missing values have been dropped, our working sample contains 1,179 respondents.^15^ Table 2 reports descriptive statistics. Average monetary commuting costs of our respondents is MXP 35 (USD 1.86) in a one-way commuting trip—with a maximum of MXP 500 (USD 26.26). In terms of self-reported income, around 52% of respondents earn MXP 15,000 (USD 787.81) on a monthly basis; 29% earn between MXP 15,000 and MXP 30,000; and 18%, more than MXP 30,000 (USD 1,575). This self-reported income is after-tax—as it is the amount respondents see in their monthly check. Respondents to our survey spend an average of 57 min in a one-way trip from home to their work place—with a maximum of 210 min (3 h and 30 min). Considering both monetary and opportunity costs, our respondents spend MXP 135 (USD 7.10) on average in a one-way commuting trip—with a maximum of MXP 806 (USD 42.33).

**Table 2:** Descriptive statistics of entire sample (1,179 respondents)

Variable	Mean	Std. Dev.	Min	Max
*Commuting costs and income*				
MC: Monetary cost of commuting (MXP)	35.55	46.29	0.00	500.00
1 if self-reported monthly income $<=$ 10K MXP$^{1}$	0.26	0.44	0.00	1.00
1 if self-reported monthly income between 10K and 15K MXP$^{1}$	0.26	0.44	0.00	1.00
1 if self-reported monthly income between 15K and 30K MXP$^{1}$	0.29	0.46	0.00	1.00
1 if self-reported monthly income > 30K MXP$^{1}$	0.18	0.38	0.00	1.00
OP: Opportunity cost of commuting (MXP/minutes)	1.82	1.09	0.52	3.64
T: Commuting time (minutes)	57.24	33.36	5.00	210.00
TC: Total cost of commuting, MC+OP*T (MXP)	135.14	110.10	2.61	806.10
*Commuting routines*				
1 if bus$^{2}$	0.41	0.49	0.00	1.00
1 if private car$^{2}$	0.26	0.44	0.00	1.00
1 if taxi/uber$^{2}$	0.05	0.21	0.00	1.00
1 if metro$^{2}$	0.22	0.41	0.00	1.00
1 if motorcycle$^{2}$	0.02	0.13	0.00	1.00
1 if bicycle$^{2}$	0.02	0.14	0.00	1.00
1 if walking$^{2}$	0.02	0.15	0.00	1.00
1 if commuting routine includes dropping off a relative	0.19	0.39	0.00	1.00
1 if commuting routine includes coordinating with colleague	0.23	0.42	0.00	1.00
1 if commuting routine includes running errands	0.44	0.50	0.00	1.00
*Office space, and job routines*				
Years in current job	4.67	6.37	0.08	39.00
1 if cubicle in room with no walls	0.39	0.49	0.00	1.00
1 if cubicle in room with walls	0.45	0.50	0.00	1.00
1 if own office	0.16	0.37	0.00	1.00
1 if private sector	0.78	0.42	0.00	1.00
1 if public sector	0.20	0.40	0.00	1.00
1 if NGO or research center	0.02	0.15	0.00	1.00
Number of average weekly job-related meetings	3.82	5.23	0.00	40.00
1 if respondent thinks his/her duties can be performed from home	0.30	0.46	0.00	1.00
Social gatherings with coworkers during last 3 months	3.91	6.22	0.00	60.00
*Respondent’s socioeconomic and demographic characteristics*				
1 if respondent is female	0.40	0.49	0.00	1.00
Respondent’s age	35.02	10.27	18.00	73.00
1 if respondent is married	0.37	0.48	0.00	1.00
Number of members of respondent’s household	3.34	1.62	1.00	15.00
1 if high school diploma or less	0.22	0.42	0.00	1.00
1 if bachelor’s degree	0.66	0.47	0.00	1.00
1 if graduate degree	0.11	0.32	0.00	1.00

$^{1}$ Based on self-reported range of monthly income, as reflected in after-tax paycheck observed by responded. Mean values of each range has been divided by 20 working days, by 8 working hours, and by 60 min. For the more than 30K range, we have assumed an *average* of 35,000 MXP.

$^{2}$ We have assigned one commuting mode to each respondent—even to multi-mode commuters. In doing so, we assigned the commuting mode in which respondents spend most of their commuting time

We offer insights on whether commuting time numbers arising from our sample are reasonable. For instance, average commuting time in our sample is 57 min. The official survey documenting commuting time and expenses in CDMX reports 56 min as average time for trips to work place (INEGI 2018)—this official number includes all jobs, not only office-related ones.^16^ The maximum commuting time in our sample is 3 h and 30 min. While this number may seem unreasonable at first, keep in mind that official numbers report that 5% of commuters spend more than 2 h (INEGI 2018), and Hess and Narteh-Yoe (2020) document that 18% of respondents to their survey in CDMX spend 3 h or more in commuting. The difference between official numbers and those reported by Hess and Narteh-Yoe (2020), we believe, originates in the data gathering strategy followed by Hess and Narteh-Yoe (2020) who have gathered data via online platforms. This strategy has likely oversampled workers with more qualifications—as digital literacy is actually necessary to answer online surveys. In this respect, numbers reported by Hess and Narteh-Yoe (2020) may reflect patterns of a population similar to ours. Thus, commuting times arising from our study fall within a feasible range of values.

In terms of commuting costs, the average total cost of one-way trip in our sample is MXP 135. These total commuting costs are calculated as the sum of monetary commuting costs (e.g. bus, and metro fees) plus total opportunity costs, which result from multiplying opportunity costs per minute times commuting minutes. Assuming 20 working days a month, this value implies costs of MXP 5,400 for 20 two-way trips. This number represents around 36% of MXP 15,000—monthly salary of a middle-income worker in Mexico (Milenio Digital 2019). Previous studies have placed this percentage in around 40% (Arredondo 2017). Thus, commuting costs documented by our study seem realistic.

**Fig. 3:**
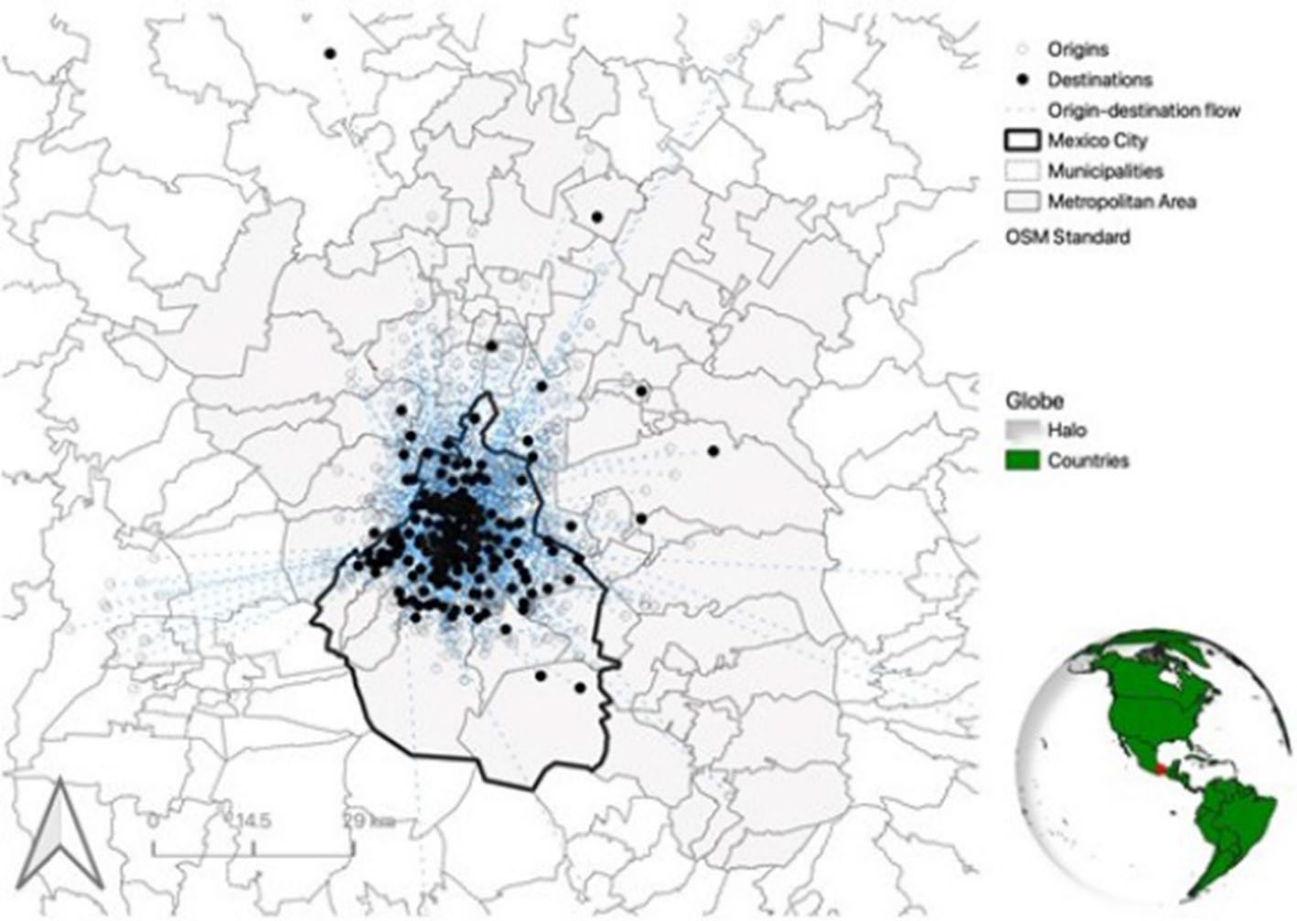
Origin-destination flows documented in this study

In addition, we have compared origin-destination flows at the municipality level arising from our survey to those documented by the official survey on travel patterns (INEGI 2018). Around 84% of the flows in our survey coincide with those of the official source, 13% flows have been oversampled by our survey, and 3% have been undersampled. Figure 3 illustrates the origin-destination flows captured by our study.

We have assigned one commuting mode to each respondent—even to multi-mode commuters. In doing so, we assigned the commuting mode in which respondents spend most of their commuting time. Table 2 reports that 41% of our respondents commute by bus; 26% commute by private car; 5% take taxi/uber; 22% commute by metro; 2% ride a motorcycle; 2% bike; and 2% walk to their jobs. In terms of commuting routines, 19% of respondents drop off a relative in their way to work; 23% of respondents coordinate with a colleague to commute to work; and 44% include running errands as part of their commuting routine.

In terms of job characteristics, Table 2 shows that our respondents have been in their job 4.67 years on average; 39% of respondents work at a cubicle in room with no walls; 45% work at a cubicle in a room with walls; and 16% work in their own office. In terms their employer’s sector, 78% of respondents work in a private company; 20% work in a public agency; and 2% work in a NGO or a research center or university. On average, respondents attend 3.82 meetings a week—with a maximum of 40 meetings a week; and 30% of respondents think that their duties can be performed from home. On average, respondents had attended 4 social gatherings with coworkers during last 3 months prior to the survey.

In terms of socioeconomic characteristics, Table 2 reports that 40% of our respondents are women; 35 years old; and 37% are married. On average, respondents household is composed by 3.34 members. In terms of education, 22% of respondents hold a high school diploma or lower degree; 66% hold a bachelor’s degree; and 11% hold a graduate degree.

## Results

### Average willingness-to-pay estimates

Table 3 reports results from five specifications. The first set of parameters results from a random parameters logit that assumes all parameters, including the price parameter, are normally distributed and uncorrelated. The second specification in Table 3 allows for correlation among random parameters. The third and fourth specifications assume that the price parameter is fixed and the rest of parameters are normally distributed—uncorrelated and correlated, respectively. The fifth specification corresponds to a conditional logit model.

**Table 3:** Conditional and random parameter logit specifications on entire sample (1,179 respondents)

Attribute	Random parameters logit				Conditional Logit
	Price parameter is assumed				
	Normally-distributed		Fixed		
	Random parameters are				
	Uncorrelated	Correlated	Uncorrelated	Correlated	
Mean parameters					
1 if not status quo alternative [1]	3.630***	3.659***	2.450***	2.355***	1.404***
	(0.193)	(0.202)	(0.144)	(0.153)	(0.0787)
Walking commuting time (hours) [2]	− 2.939***	− 3.017***	− 2.284***	− 2.272***	− 1.398***
	(0.177)	(0.180)	(0.153)	(0.155)	(0.0914)
1 if infrastructure for bike parking [3]	0.707***	0.764***	0.621***	0.723***	0.437***
	(0.0672)	(0.0865)	(0.0560)	(0.0734)	(0.0434)
Amount subtracted from monthly paycheck [4]	− 2.492***	− 2.451***	− 1.414***	− 1.388***	− 1.080***
(thousand MXP)	(0.121)	(0.122)	(0.0540)	(0.0560)	(0.0385)
Standard deviation of parameters					
1 if not status quo alternative [1]	3.072***		2.686***		
	(0.169)		(0.134)		
Walking commuting time (hours) [2]	1.785***		1.800***		
	(0.297)		(0.227)		
1 if infrastructure for bike parking [3]	0.818***		0.336*		
	(0.157)		(0.191)		
Amount subtracted from monthly paycheck[4]	1.664***				
(thousand MXP)	(0.106)				
Elements of lower-triangular matrix L, where covariance matrix of random coefficients is $V = L^{'}L$					
L11		3.077***		2.770***	
		(0.248)		(0.187)	
L21		0.531**		0.266	
		(0.260)		(0.306)	
L31		− 0.0159		− 0.296**	
		(0.136)		(0.0981)	
L41		− 0.277**			
		(0.133)			
L22		0.246		1.525***	
		(0.258)		(0.357)	
L32		1.083***		− 0.0589	
		(0.121)		(0.122)	
L42		− 0.0136			
		(0.114)			
L33		0.269**		0.449**	
		(0.125)		(0.213)	
L43		1.670***			
		(0.1000)			
L44		0.374**			
		(0.166)			
Observations	14148	14148	14148	14148	14148
Respondents	1179	1179	1179	1179	1179
Log-likelihood	− 3659.2	− 3653.3	− 3810.7	− 3806.5	− 4465.4
AIC	7334.3	7334.7	7635.4	7633.1	8938.9
BIC	7394.8	7440.5	7688.3	7708.6	8969.1

Standard errors in parentheses

*p < 0.10, **p < 0.05, ***p < 0.001

The signs of parameters associated with each attribute are as expected across all five econometric specifications. The parameter associated with a non-status quo alternative-specific variable is positive—indicating a preference for teleworking alternatives over the status quo alternative of working from office. The commuting time parameter is negative—indicating disutility from time spent in commuting. The parameter capturing preferences for infrastructure to park bikes is positive—indicating a preference for presence of infrastructure to park a bike. The price parameter is negative—indicating a disutility from cuts in salary. All parameters are significant with p-value lower than 0.001.

Unobserved heterogeneity in preferences is confirmed by standard deviation and correlation estimates arising from all four random parameters specifications reported in Table 3. When it comes to standard deviation of parameters, all but one specification yield all four standard deviation parameters as statistically significant. The exception is the non-statistically significant standard deviation parameter for commuting time (L22 in Table 3) arising from the specification that assumes price parameter is random and all random parameters are correlated. When it comes to correlation parameters, estimates depend on whether price parameter is assumed fixed or random. The specification that assumes a random price parameter yields statistically significant correlation between preferences for non-status quo alternative and commuting time (L21), non-status quo alternative and price (L41), commuting time and infrastructure for bike parking (L32), and price and infrastructure for bike parking (L43). The specification that assumes a fixed price parameter yields only one statistically significant correlation parameter—between infrastructure for bike parking and non-status quo alternative (L31).

Notice that the random parameter specification that assumes all parameters are random yields statistically significant a correlation reflecting counterintuitive patterns. In particular, the positive correlation between preferences for commuting time and non-status quo alternative-specific variable ($L21=0.531$) is counterintuitive because we would expect that higher preferences for teleworking are associated with lower preferences for commuting time—i.e. we would expect a negative correlation.^17^

The bottom panel of Table 3 reports Akaike Information Criterion (AIC) and Bayesian Information Criterion (BIC) for each specification. According to both criteria, all four random parameters logit specifications outperform the conditional logit model. Also according to both criteria, specifications assuming that preferences for price are random outperform specifications assuming a fixed price parameter. From among the two models assuming price parameter is normally distributed, the one with uncorrelated parameters slightly outperforms the one assuming correlation—with an AIC of 7334.3 versus 7334.7, and a BIC of 7394.8 versus 7440.5.

Table 4 reports willingness to pay (WTP) estimates—expressed in monthly thousand Mexican pesos (MXP)—arising from the five specifications in Table 3. The first set of numbers refers to average WTP for not remaining in status quo—i.e. WTP for teleworking—, and lower and upper bounds reflecting a 95% confidence interval. The second set of estimates refer to WTP for one hour of walking commuting—i.e. the value respondents’ assign to one hour of commuting. The negative sign of this estimate reflects that respondents require compensation for bearing one hour of commuting. The third set of estimates refer to the WTP for infrastructure to park bikes.

**Table 4:** Willingness to pay (WTP) and 95% confidence intervals (monthly thousand MXP) resulting from specifications reported in Table 3

WTP for	Random parameters logit				Conditional Logit
	Price parameter is assumed				
	Normally-distributed		Fixed		
	Random parameters are				
	Uncorrelated	Correlated	Uncorrelated	Correlated	
*Not remaining in status quo*	1.46	1.49	1.73	1.70	1.30
Lower Bound	1.33	1.36	1.57	1.51	1.18
Upper Bound	1.58	1.63	1.90	1.89	1.42
*One hour of walking commuting time*	− 1.18	− 1.23	− 1.61	− 1.64	− 1.29
Lower Bound	− 1.31	− 1.38	− 1.80	− 1.84	− 1.45
Upper Bound	− 1.05	− 1.09	− 1.42	− 1.43	− 1.14
*Bike parking infrastructure*	0.28	0.31	0.44	0.52	0.40
Lower Bound	0.23	0.24	0.36	0.41	0.32
Upper Bound	0.34	0.39	0.52	0.63	0.49

We highlight two features of Table 4. First, while point estimates of WTP differ across the five specifications, their 95% confidence intervals largely intersect. This feature holds for WTP of all three attributes under consideration. The second feature of interest in Table 4 is that, from among the four random parameters specifications, the most conservative average estimates are delivered by the specification that assumes prices parameter is normally distributed and no correlation among random parameters.

Our preferred model is the random parameters specification that assumes price is normally distributed and no correlation among random parameters. This is so when considering together three features. First, the relative statistical performance indicated by both AIC and BIC—these penalized-likelihood together suggest that the assumption of correlation among parameters does not improve statistical performance of econometric specifications. Second, models assuming correlation among random parameters yield insignificant standard deviation for the commuting time parameter, and a counterintuitive sign for the association between commuting time parameter and non-status quo parameter. Third, the most conservative point WTP estimates are delivered by the model assuming price parameter is normally distributed and no correlation among random parameters—and 95% confidence intervals of these point estimates intersect with those of the other four models.

Thus, our preferred estimation of average monthly WTP for a teleworking alternative is MXP 1,460 (USD 76.68). The average monthly value of one hour of commuting time is MXP 1,180 (USD 61.97). And the average monthly WTP for bike parking infrastructure is MXP 280 (USD 14.70). The monthly WTP for one hour of commuting time must be interpreted as the value assigned by the respondent to 16 h of commuting time over a given month. This interpretation results from the phrasing of the scenario under valuation. As the respondent ponders teleworking two days a week in exchange for a discount to his/her monthly check, if teleworking is chosen then the respondent saves him/herself eight two-way trips during a given month. Thus the hourly value is obtained by dividing MXP 1,180 over 16, which yields MXP 73.75 (USD 3.87).

### Observed heterogeneity in willingness-to-pay estimates

In addition to estimation of average WTP in the context of unobserved heterogeneity, we have explored the presence of observed heterogeneity. In doing so, we have followed a two-stage procedure suggested by Campbell (2007). First, he uses coefficients from random parameter models to estimate WTP values for each respondent, and then runs OLS specifications on individual WTP as a function of individual-specific characteristics.^18^ Accordingly, we have first estimated individual-specific WTP based coefficients delivered by the random parameters specification that assumes price parameter is normally distributed and no correlation among random parameters—as reported in Table 3. Then, we have modeled WTP for each attribute separately via OLS, and also simultaneously via Seemingly Unrelated Regressions (SUR). These models have been estimated on our entire sample of respondents, and on three trimmed samples that have excluded lower and upper tails—1%, 5%, and 10% tails, respectively.

This two-stage exploration has delivered few factors that can consistently be associated with observed heterogeneity across sub-samples. As an illustration, Table 5 reports six OLS specifications on individual WTP for teleworking alternatives—i.e. WTP for not remaining in status quo. These models have been estimated on a sub-sample that excludes lower and upper tails at 10%. Table 5 illustrates results when sequentially accounting for variables reflecting job-related characteristics, commuting-related characteristics, and socioeconomic and demographic characteristics—these variables have been selected based on factors identified in section 2.3. Specification (I) models individual WTP as a function of job-related characteristics, including (i) whether the respondent thinks his/her duties can be performed from home; (ii) average weekly job-related meetings; (iii) years in current job; (iv) type of office space at employer’s premises—with own office serving as reference category; v) whether respondent works in private sector—with public sector and NGO serving as reference category; and (vi) whether respondent has attended zero social gatherings in last months three months—zero is the value corresponding to the 25th percentile of social gatherings attended. Specification (II) adds commuting-related characteristics to specification (I). These characteristics include (i) commuting mode—bus, private car, taxi/uber, and metro, with walking or motor/bicycle as reference category;^19^ (ii) whether commuting time is larger than 50 min—which is the sample median; (iii) whether total commuting costs are larger than MXP 102.65 (USD 5.39)—which is the sample median; (iv) variables capturing commuting routines—dropping off a relative, coordinating with a colleague, and running errands. Specification (III) adds socioeconomic and demographic characteristics to specification (II). These characteristics include (i) whether respondent is female; (ii) respondent’s age; (iii) whether respondent is married; (iv) people living in respondent’s dwelling; (v) respondent’s education—bachelor, and graduate degree, with high school or less serving as reference category; and (vi) whether respondent’s self-reported monthly income is more than MXP 15,000 (USD 787.82)—this category includes around 48% of our respondents, and is based on a threshold used to define who is middle-income class in Mexico (Milenio Digital 2019). Specifications (IV) to (VI) add, respectively, interactions between income and commuting time categories, between commuting mode and commuting time, and between income and commuting mode.

**Table 5:** Ordinal-Least Squared specifications on individual WTP for teleworking alternatives. Estimates are obtained from a Random Parameters Logit that assumes price parameter is normally distributed and no correlation among random parameters (reported in Table 3)—10% lower and upper tails excluded

Characteristic	(I)	(II)	(III)	(IV)	(V)	(VI)
*Job-related characteristics*						
1 if respondent thinks all his/her duties can be performed from home	0.0212	0.0157	0.00987	0.00984	0.0145	0.0146
	(0.0748)	(0.0752)	(0.0757)	(0.0757)	(0.0757)	(0.0758)
Average weekly job-related meetings	− 0.00137	− 0.00255	− 0.00182	− 0.00173	− 0.00195	− 0.00213
	(0.00735)	(0.00763)	(0.00783)	(0.00785)	(0.00779)	(0.00785)
Years in current job	− 0.00698	− 0.00680	− 0.00698	− 0.00697	− 0.00749	− 0.00741
	(0.00644)	(0.00653)	(0.00724)	(0.00724)	(0.00720)	(0.00724)
1 if cubicle in room with no walls	− 0.126	− 0.127	− 0.142	− 0.144	− 0.149	− 0.147
	(0.0997)	(0.101)	(0.102)	(0.102)	(0.102)	(0.103)
1 if cubicle in room with walls	− 0.00885	− 0.0121	− 0.0202	− 0.0211	− 0.0233	− 0.0221
	(0.0986)	(0.100)	(0.101)	(0.101)	(0.101)	(0.102)
1 if private sector	0.0276	0.0368	0.0263	0.0276	0.0274	0.0261
	(0.0840)	(0.0855)	(0.0869)	(0.0872)	(0.0868)	(0.0871)
1 if respondent attended zero social gatherings in last three months	− 0.145**	− 0.164**	− 0.141*	− 0.143*	− 0.136*	− 0.136*
	(0.0716)	(0.0726)	(0.0757)	(0.0757)	(0.0762)	(0.0767)
*Commuting − related characteristics*						
1 if bus		0.348**	0.340**	0.338**	0.293*	0.340
		(0.148)	(0.148)	(0.149)	(0.167)	(0.220)
1 if private car		0.320**	0.330**	0.328**	0.480**	0.504**
		(0.155)	(0.155)	(0.156)	(0.178)	(0.241)
1 if taxi/uber		0.253	0.255	0.255	0.0796	0.171
		(0.205)	(0.209)	(0.209)	(0.258)	(0.322)
1 if metro		0.320**	0.312**	0.309**	0.358**	0.422*
		(0.153)	(0.153)	(0.153)	(0.176)	(0.236)
1 if commuting time $>50$ min		0.0438	0.0445	0.0179	0.191	0.205
		(0.0777)	(0.0808)	(0.0985)	(0.398)	(0.398)
1 if total cost of commuting > MXP 102.65		0.0893	0.0667	0.0666	0.0527	0.0584
		(0.0819)	(0.0965)	(0.0965)	(0.0970)	(0.0985)
1 if commuting routine includes dropping off a relative		− 0.102	− 0.0896	− 0.0889	− 0.0676	− 0.0663
		(0.0899)	(0.0903)	(0.0903)	(0.0900)	(0.0900)
1 if commuting routine includes coordinating with colleague		− 0.0320	− 0.0394	− 0.0436	− 0.0498	− 0.0503
		(0.0775)	(0.0783)	(0.0783)	(0.0787)	(0.0790)
1 if commuting routine includes running errands		− 0.00655	− 0.00653	− 0.00537	− 0.00150	− 0.00192
		(0.0681)	(0.0689)	(0.0691)	(0.0690)	(0.0698)
*Socioeconomic and demographic characteristics*						
1 if respondent is female			0.00986	0.00994	0.0114	0.0119
			(0.0729)	(0.0730)	(0.0726)	(0.0729)
Respondent’s age			0.000235	0.000318	0.000521	0.000462
			(0.00444)	(0.00446)	(0.00447)	(0.00451)
1 if respondent is married			− 0.0777	− 0.0787	− 0.0760	− 0.0748
			(0.0823)	(0.0824)	(0.0822)	(0.0823)
People living at respondent’s dwelling			0.0129	0.0122	0.00944	0.00874
			(0.0209)	(0.0210)	(0.0209)	(0.0211)
1 if bachelor’s degree			0.0143	0.0128	0.0196	0.0194
			(0.0868)	(0.0867)	(0.0868)	(0.0870)
1 if graduate degree			− 0.130	− 0.130	− 0.139	− 0.140
			(0.143)	(0.143)	(0.144)	(0.144)
1 if self-reported monthly income > MXP 15,000			0.0692	0.0381	0.00635	0.0841
			(0.0870)	(0.115)	(0.116)	(0.275)
*Interactions*						
1 if self-reported monthly income > MXP 15,000 X				0.0603	0.133	0.140
1 if commuting time $>50$ min				(0.136)	(0.140)	(0.144)
1 if bus X					− 0.0783	− 0.0976
1 if commuting time $>50$ min					(0.406)	(0.407)
1 if private car X					− 0.457	− 0.475
1 if commuting time $>50$ min					(0.417)	(0.418)
1 if taxi/uber X					0.211	0.175
1 if commuting time $>50$ min					(0.489)	(0.491)
1 if metro X					− 0.255	− 0.276
1 if commuting time $>50$ min					(0.413)	(0.415)
1 if bus X						− 0.0866
1 if self − reported monthly income > MXP 15,000						(0.304)
1 if private car X						− 0.0527
1 if self-reported monthly income > MXP 15,000						(0.312)
1 if taxi/uber X						− 0.148
1 if self-reported monthly income > MXP 15,000						(0.399)
1 if metro X						− 0.124
1 if self-reported monthly income > MXP 15,000						(0.311)
Intercept	1.537***	1.199***	1.165***	1.184***	1.174***	1.133***
	(0.123)	(0.184)	(0.245)	(0.245)	(0.251)	(0.274)
Observations	945	945	945	945	945	945
R-squared	0.00303	0.00567	0.00170	0.000819	0.00391	0.000183
Log-likelihood	− 1364.9	− 1359.1	− 1357.4	− 1357.3	− 1353.7	− 1353.6
AIC	2745.7	2752.1	2762.7	2764.5	2765.5	2773.2
BIC	2784.5	2834.6	2879.2	2885.8	2906.2	2933.3

Standard errors in parentheses

*p < 0.10, **p < 0.05, ***p < 0.001

The general message from Table 5 is that our two-stage strategy identifies few individual-specific factors that can be consistently associated with individual WTP estimates for teleworking—and this finding holds for WTP for bike parking infrastructure, and value of commuting time as well.^20^ One of those few factors is captured by the binary variable reflecting whether a respondent has attended zero job-related social gatherings during the last three months. Taking coefficients from specification (II) as illustrative case, respondents that do not attend social gatherings report a WTP for teleworking lower by around MXP 164 (USD 8.61) than the conditional average WTP of MXP 1,199 (USD 62.97) in the sub-sample—recall that this average does not include lower and upper tails at 10%. This finding may be interpreted as reflecting that office workers deemed as less socially-engaged report to be less interested on teleworking alternatives.

Table 5 illustrates that our two-stage strategy identifies commuting mode as the other factor that can be associated with differences in WTP for teleworking. Taking as reference the category that pools respondents that walk or use motor/bicycle for commuting purposes, respondents commuting by bus report higher-than-average WTP by around MXP 348 (USD 18.27). Those commuting by private car report a higher WTP by MXP 320 (USD 16.81), a WTP shared by those commuting by metro.

As Table 5 illustrates, not a single socioeconomic or demographic characteristic turns out to be associated with heterogeneity in WTP—see specification (III). Similarly, no statistical gains are obtained when adding interactions between commuting mode and income, or commuting mode and commuting time —statistically insignificant coefficients from these interactions are reported in columns (IV), (V), and (VI) in Table 5.

Indeed, it is difficult to disentangle partial correlation coefficients when collinearity is at place, which is likely to be the case in our application. In particular, a relatively large association is expected among income, commuting mode, commuting time, and commuting costs. At the same time, these factors are associated with revealed preferences—i.e. real-life decisions such as location of home with respect to job place—, and therefore we are interested in exploring whether these factors are associated with differences in stated WTP—as it can help us evaluate realism and reliability of our estimates. For instance, we would expect that respondents spending relatively less commuting time are those stating higher disutility from one hour of commuting.

Thus we have implemented an alternative strategy to explore observed heterogeneity in WTP estimates. In doing so, we have repeated the five econometric specifications described in Table 3. This time instead of analyzing the entire sample, we have analyzed sub-samples defined based on categories of income, commuting mode, commuting time, and total commuting costs, and intersections of categories across these variables.^21^ Findings resulting from these five specifications on each sub-sample are reported in Tables 8, 9, and 10. As in the case of the entire sample, AIC and BIC criteria suggest that, across sub-samples, the best statistical fit is delivered by the specification that assumes price parameter is normally distributed and random parameters are uncorrelated.

Thus, discussion of heterogeneity of WTP focuses on estimates arising from a random parameters logit that assumes price is normally distributed and no correlation among random parameters—as reported in Tables 8, 9, and 10. Accordingly, Table 6 reports average estimates of WTP by sub-sample based on categories of (i) self-reported income—below and above MXP 15,000 (USD 787.82), respectively—; (ii) commuting time—below and above 50 min, respectively—; and (iii) total commuting costs—below and above MXP 102.65 (USD 5.39), respectively. Thresholds defining these categories have been set based on sample median values. Accordingly, the sub-sample with less (more) than MXP 15,000 encompasses 620 respondents (559 respondents); the sub-sample that spends less (more) than 50 min in commuting includes 606 respondents (573 respondents); and the sub-sample that with total commuting costs below (above) MXP 102.65 includes 588 respondents (591 respondents).

**Table 6:** Willingness to pay (WTP) and 95% confidence intervals (monthly thousand MXP) resulting from random parameter logit specifications that assume price parameter is normally distributed and no correlation among random parameters—reported in Tables 8, 9, and 10

WTP for	Income (MXP)		Commuting time (minutes)		Total commuting costs (MXP)	
	< 15,000	> 15,000	< 50	> 50	< 102.65	> 102.65
Not remaining in status quo	1.26	1.65	1.45	1.42	1.29	1.56
Lower Bound	1.1	1.45	1.27	1.26	1.11	1.39
Upper Bound	1.43	1.86	1.64	1.6	1.48	1.73
One hour of walking commuting time	$-$0.88	$-$1.52	$-$1.55	$-$0.83	$-$1.05	$-$1.22
Lower Bound	$-$1.04	$-$1.77	$-$1.79	$-$0.98	$-$1.24	$-$1.39
Upper Bound	$-$0.72	$-$1.29	$-$1.33	$-$0.68	$-$0.87	$-$1.05
Bike parking infrastructure	0.29	0.27	0.31	0.25	0.27	0.29
Lower Bound	0.22	0.18	0.23	0.18	0.19	0.21
Upper Bound	0.36	0.36	0.4	0.33	0.35	0.36

Table 6 illustrates that, as economic theory suggests, (i) WTP for teleworking varies depending on income and commuting time; and (ii) stated value of commuting time varies by category of commuting time. It also illustrates that WTP for bike parking infrastructure remains fairly homogeneous across income and commuting time categories.

In particular, when it comes to differences across income categories, Table 6 shows that respondents with income above MXP 15,000 (USD 787.82) report a WTP of MXP 1,650 (USD 86.65) for teleworking, and those with income lower than MXP 15,000 report a WTP of MXP 1,260 (USD 66.17)—we deem the difference of these point estimates as different from zero because their 95% confidence intervals do not overlap. Similarly, respondents with income above MXP 15,000 report a monthly value for one hour of commuting of MXP 1,520 (USD 79.83), and respondents with income below MXP 15,000 report a WTP of MXP 880 (USD 46.21)—and no overlap is observed between 95% confidence intervals of these point estimates. WTP for bike parking infrastructure is fairly homogeneous across income categories—MXP 290 (USD 15.23) on a monthly basis when income is below MXP 15,000, and MXP 270 (USD 14.18) on a monthly basis when income is above MXP 15,000.

When it comes to WTP estimates by sub-samples based in commuting time, Table 6 illustrates heterogeneity in value of one hour of commuting time. Respondents spending less than 50 min in commuting assign a monthly value of MXP 1,550 (USD 81.41) to one hour of commuting, and those spending more than 50 min assign a value of MXP 830 (43.59)—95% confidence interval of these point estimates do not overlap. WTP for teleworking and bike parking infrastructure remain fairly homogeneous across commuting time categories.

Table 6 shows a lack of heterogeneity in WTP estimates when it comes to comparisons across sub-samples based in total commuting costs. Total commuting costs have been calculated as the addition of monetary costs of commuting and opportunity costs (calculated as opportunity costs per hour times hours spent commuting). Given that Table 6 documents heterogeneity in stated value of commuting time by categories of income and commuting time, the lack of heterogeneity across total commuting costs imply that respondents falling in different categories of income and commuting time are *reshuffled* when categorizing by total commuting costs—i.e. wealthier respondents that spend less than 50 min in commuting are allocated within the same category of commuting costs than less wealthy respondents that spend more than 50 min in commuting. This is not surprising as people take into consideration their wages when deciding location of their home with respect to their job place, commuting mode, and commuting time, which implies that two people may pay similar total commuting costs even if commuting time and wage differs.

**Fig. 4:**
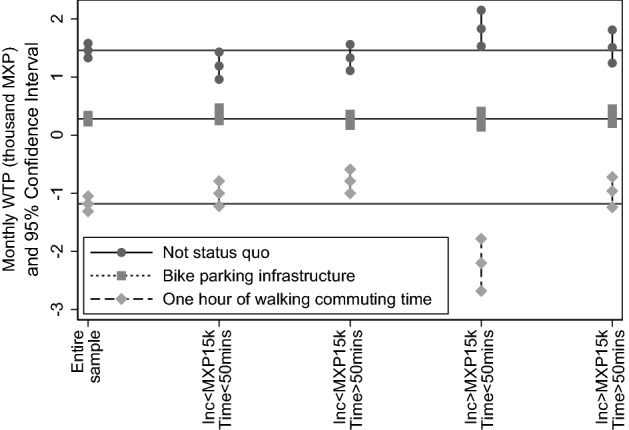
Confidence interval of monthly WTP (thousand MXP) for each attribute in our discrete choice experiments—resulting from estimating Random Parameter Logit (assuming price parameter normally distributed and no correlation among random parameter) on subsamples resulting from interacting income and commuting time —estimated coefficients are reported in Table 11

For purposes of our analysis, the implication is that it is worth exploring whether WTP estimates are heterogeneous across sub-samples of respondents resulting from combining categories of income and commuting time. Thus, we have explored whether observed heterogeneity can be documented by estimating random parameter logit specifications on four sub-samples—respondents with income below MXP 15,000 and spending less than 50 min in commuting; with income below MXP 15,000 and spending more than 50 min in commuting; with income above MXP 15,000 and spending less than 50 min in commuting; and with income above MXP 15,000 and spending more than 50 min in commuting. Figure 4 illustrates WTP estimates for each sub-sample. These estimates result from a random parameter logit that assumes price parameter is normally distributed and no correlation among random parameters —parameter estimates are reported in Table 11.

We highlight three features illustrated by Fig. 4. First, income drives differences in WTP for teleworking—i.e. for not remaining in status quo. Focusing in respondents that spend less than 50 min in commuting, those with income higher than MXP 15,000 report a monthly WTP of MXP 1,830 (USD 96.11), and those with income higher than MXP 15,000 report a monthly WTP of MXP 1,190 (USD 62.50)—recall that the average WTP in the entire sample is MXP 1,460 (USD 76.68). That is, from among those that have revealed that they prefer lower commuting times, wealthier respondents express a higher stated demand for teleworking than average, and less wealthy ones express lower demand than average. When focused on respondents spending more than 50 min in commuting, differences in income are not associated with differences in WTP for teleworking—i.e. respondents in these sub-samples report a similar WTP than the average of the entire sample.

A second feature that we highlight from Fig. 4 is that the interaction between income and commuting time drives differences in stated value of commuting time. The sub-sample with the lowest disutility from commuting time (MXP 790; USD 41.49) is the one composed by respondents with less than MXP 15,000 that spend more than 50 min in commuting. The sub-sample with the most disutility from commuting time (MXP 2,200; USD 115.54) is the one composed by respondents with more than MXP 15,000 that spend more less than 50 min in commuting—recall that the average disutility from commuting time is MXP 1,180 (USD 61.97) for the entire sample. For the rest of sub-samples, we cannot reject the null hypothesis that differences with respect to the average value are zero.

A third feature that we highlight from Fig. 4 is that WTP for bike parking infrastructure is fairly homogeneous across all four sub-samples —i.e. neither income nor commuting time seem to be associated with differences in WTP for bike parking infrastructure.

We have also explored heterogeneity in WTP by analyzing sub-samples based on commuting mode—bus, private car, taxi/uber, metro, and motor/bicycle or walking. The bus sub-sample encompasses 486 respondents; the private car sub-sample, 308 respondents; the taxi/uber sub-sample, 55 respondents; the metro sub-sample, 256 respondents; and the motor/bicycle or walking sub-sample, 74 respondents. Table 7 reports WTP estimates on these sub-samples—estimates arise from random parameter logit specifications reported in Tables 12, 13, and 14.

**Table 7:** Willingness to pay (WTP) and 95% confidence intervals (monthly thousand MXP) resulting from random parameter logit specifications that assume price parameter is normally distributed and no correlation among random parameters—reported in Tables 12, 13, and 14

WTP for	Commuting mode				
	Bus	Private car	Taxi/uber	Metro	Motor- or bicycle, or walking
Not remaining in status quo	1.33	1.59	1.31	1.49	1.18
Lower Bound	1.14	1.34	0.84	1.25	0.68
Upper Bound	1.52	1.85	1.86	1.76	1.66
One hour of walking commuting time	$-$0.85	$-$1.55	$-$1.08	$-$1.24	$-$1.63
Lower Bound	$-$1.02	$-$1.87	$-$1.71	$-$1.56	$-$2.22
Upper Bound	$-$0.69	$-$1.26	$-$0.56	$-$0.95	$-$1.04
Bike parking infrastructure	0.27	0.23	0.26	0.31	0.42
Lower Bound	0.20	0.10	0.03	0.19	0.14
Upper Bound	0.35	0.36	0.56	0.44	0.72

The general message from Table 7 is that while average estimates of WTP may differ across commuting mode sub-samples, 95% confidence intervals largely intersect with the 95% confidence interval of WTP estimates obtained from the entire sample. This finding holds when it comes to WTP teleworking and bike parking infrastructure, and for the case of value of commuting time there is one exception—the bus sub-sample reports the lowest value of commuting time across commuting modes—MXP 850 (USD 44.64).

**Fig. 5:**
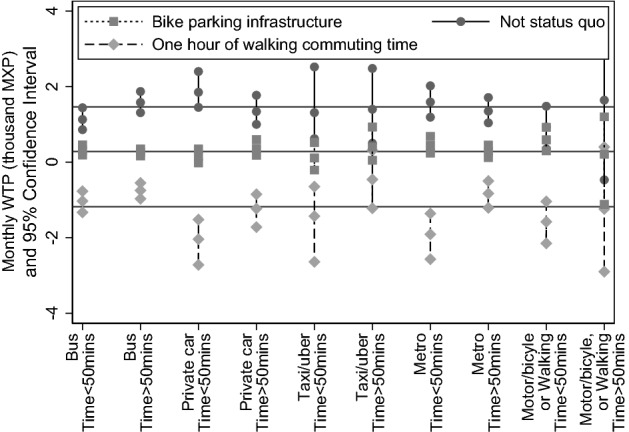
Confidence interval of monthly WTP (thousand MXP) for each attribute in our discrete choice experiments—resulting from estimating Random Parameter Logit (assuming price parameter normally distributed and no correlation among random parameter) on subsamples resulting from interacting commuting mode and commuting time—estimated coefficients are reported in Table 15

Given that findings in Table 5 suggest that commuting mode plays a role when simultaneously controlling for a number of individual-specific variables, we have explored further potential differences in WTP across commuting mode by estimating random parameter specifications on sub-samples arising from intersection between commuting mode categories and commuting time—coefficients from these specifications are reported in Table 15. Figure 5 reports WTP estimates arising from those specifications. We highlight four features from this figure. First, one sub-sample stands out in terms of both WTP for teleworking and value of commuting time. This sub-sample is composed by respondents that commute in private car and spend less than 50 min in commuting. These respondents report higher than average WTP for teleworking—MXP 1,850 (USD 97.16) versus MXP 1,460 (USD 76.68)—, and an average value of commuting time almost twice the average value in the sample—MXP 2,040 (USD 107.14) versus MXP 1,180 (USD 61.97).

A second feature that we highlight from Fig. 5 is that two sub-samples report lower than average WTP for teleworking. The first sub-sample is composed by respondents that commute by bus and spend less than 50 min –their WTP is MXP 1,130 (USD 59.34). The second sub-sample is composed by respondents that spend less than 50 min and walk or ride motor/bicycle to their job—their WTP is MXP 930 (USD 48.84).

We also highlight two other sub-samples that report lower- and higher-than-average values of commuting time, respectively. On one hand, the sub-sample that commutes by bus and spends more than 50 min report a lower-than-average value of MXP 750 (USD 39.39). On the other hand, the sub-sample that commutes by metro and spends less than 50 min report a higher-than-average value of MXP 1,910 (USD 100.31).

A fourth highlight from Fig. 5 is the sub-sample that reports a higher-than-average WTP for bike parking infrastructure. This sub-sample is composed by respondents that spend less than 50 min in their commute and either walk or ride motor/bicycle to their job—their WTP is MXP 590 (USD 30.98), which is more than twice the average value of MXP 280 (USD 14.70).

## This paper’s estimates in perspective

### Comparison against revealed preferences values and previous studies

The most novel contribution of this study is the estimation of stated WTP of office workers in CDMX for teleworking from a near-home shared office two days a week. The average WTP for teleworking is estimated at around (2019) MXP 1,460 (USD 76.68) on a monthly basis.

As with any stated preferences study, there is the possibility that our estimates are biased due to the hypothetical feature of the scenario presented to respondents. To evaluate whether our estimates fall within a reasonable range of values, we present a number of comparisons with respect to values revealed in real markets. For instance, average WTP for teleworking represents 8.40% of average monthly income of respondents in our sample. It also represents 33.76% of average total commuting costs of 16 two-way trips to their current office—16 is the number of two-way trips involved in the teleworking scenario under analysis. Considering that transportation costs in Mexico City can consume up to a third of a salary of a minimum-wage worker, and as much as 40% of an annual middle-income class salary (Arredondo 2017; Guerra 2017), that WTP for teleworking represents around 33.76% of commuting costs seems reasonable. Our WTP estimates can also be compared to market prices of renting a shared office in 2019. WeWork charged around MXP 4,500 per month (USD 236.34) per desk space in Mexico City in 2019.^22^ This rate is equivalent to MXP 225 (USD 11.82) per working day. Our average WTP translates into a daily rate of MXP 182.5 (USD 9.58); and our highest WTP translates into MXP 228 (USD 11.97)—just at Wework’s rate.

When it comes to variation in WTP for teleworking, wealthier respondents with shorter-than-the-median commuting time report the highest WTP for teleworking. This finding holds for sub-samples for which wealth is approximated either as monthly income or through private car as main commuting mode. In particular, respondents with monthly income above MXP 15,000 (USD 787.82)—an amount that includes around 48% of our respondents, and is used as threshold to define who is middle-income class in Mexico (Milenio Digital 2019)—that spend less than 50 min in commuting —the median commuting time in our sample—report a WTP for teleworking of MXP 1,830 (USD 96.11). Respondents within the same commuting time category that commute by private car report a WTP for teleworking of MXP 1,850 (USD 97.16).

Less wealthy respondents with shorter-than-the-median commuting time report the lowest WTP for teleworking. Respondents spending less than 50 min in commuting and with monthly income lower than MXP 15,000 report a WTP for teleworking of MXP 1,190 (USD 62.50). Similarly, respondents within the same commuting time category that commute by bus report a WTP for teleworking of MXP 1,130 (USD 59.34). Respondents within the same commuting time category that commute by riding a motor/bicycle or by walking report a WTP for teleworking of 930 (USD 48.48).

That less wealthy office workers spending shorter-than-the-median commuting time report lower WTP for teleworking is consistent with economic theory as they i) have less income to afford this service; and ii) benefit less from teleworking—in comparison to workers commuting longer-than-the-median time. The higher WTP for teleworking reported by wealthier office workers with shorter commutes is explained by the value of their time. Living relatively close to their jobs saves them commuting time, and the opportunity of teleworking would save them even more time. This intuition implies that these respondents would also report a relatively higher value of commuting time—which is the case.

The average value of one hour of commuting time is estimated at MXP 73.35 (USD 3.87). To ponder the potential presence of bias arising from the hypothetical nature of our scenario, we compare this average value to numbers inferred from real market decisions in similar contexts. In particular, Garsous et al. (2019) report that the introduction of cable cars in La Paz, Bolivia, has translated into a daily reduction of 9 min in commuting time and an average net benefit of USD 0.58 per commute. These numbers are equivalent to an hourly value of ($(60\ mins /9\ mins)*USD\ 0.58=$) USD 3.87, which is identical to the average value arising from our hypothetical scenario. The estimated average value of one hour of commuting time can also be compared to the average total commuting cost of a one-round trip in our sample—MXP 135.14 (USD 7.10). A recent review of values of travel time in Europe, covering 3,109 monetary valuations from 389 studies, reports estimates of commuting time covering a range of values from (2019) USD 9.93 to USD 14.75 (Wardman et al. 2016).^23^ While our average estimates fall below these numbers, we highlight that our highest estimate is close to Europe’s lowest value (USD 7.22 versus USD 9.93). In general, average estimates of value of commuting time reported in this paper seem reasonable and realistic.

The highest estimated value of one hour of commuting time is almost twice as large as the mean value—MXP 137.50 (USD 7.22). This value has been reported by respondents with shorter-than-the-median commuting time that make more than MXP 15,000 on a monthly basis. This higher-than-average value is consistent with what these respondents have already revealed in the housing market—i.e. that it is important for them to live relative close to their jobs. Within the same commuting time category, respondents that commute by private car report a WTP of MXP 127.50 (USD 6.69). On the other hand, respondents that make less than MXP 15,000 on a monthly basis and spend more than 50 min in commuting report the lowest value of one hour of commuting time—MXP 49.38 (USD 2.59). Similarly, respondents within the same commuting time category that commute by bus report a value of MXP 46.87 (USD 2.46).

Taking WTP for teleworking and value of commuting time, we can learn the commuting time at which working from a shared office becomes unappealing. In particular, if an office worker has to commute one hour to a shared office then his/her WTP is calculated by subtracting MXP 1,180 to MXP 1,460. That is, MXP 280 (USD 14.07) is a respondent’s WTP for teleworking 2 days a week from an office that is one hour away from his/her home. Considering that average commuting time is 57 min, it seems reasonable that our respondents’ average WTP for teleworking is almost out-weighted by a one hour of commuting.

A third set of estimates reported in this study refers to WTP for bike parking infrastructure. The average value is estimated at MXP 280 (USD 14.07) on monthly basis. This number reflects benefits that office workers would experience with infrastructure to safely park their bikes if they commuted by bike. To put context to this estimate, we point out that it is twice as much to the highest value of one hour of commuting time estimated in this study. That is, our respondents’ monthly benefits from infrastructure to park safely their bikes double the value of one hour of commuting time of the wealthier workers. While WTP for bike parking infrastructure is fairly homogeneous across sub-samples of respondents, we have identified one sub-sample for which bike parking infrastructure represents more than twice as much the average value (MXP 590, USD 30.98). This sub-sample is composed by respondents that either walk or bike or ride a motorcycle to their job, and spend less than 50 min in their commute. Indeed, these office workers are the ones that can benefit the most from bike parking infrastructure.

### Informing public policy conversations

The range of specific public policies that our estimates can inform is wide because measures that enable teleworking and/or potentially decrease commuting time fall within a number of domains. In this section, we illustrate how our estimates may inform debates about policies dealing with transportation, housing, and labor.

As provision of Bus Rapid Transit (BRT) systems decreases commuting time, values of commuting time in this paper provide estimates of benefits from transportation policies. In 2005, a BRT system was launched in Mexico. While estimates of reductions in commuting time brought by this BRT system have been provided by Wöhrnschimmel et al. (2008), there is no previous estimation of how much these savings in time translate into monetary terms—a gap explained by, among other factors, a lack of estimates of value of commuting time for Mexico City. In particular, commuters using *minibuses*^24^ experienced a reduction in commuting time from 72 to 58 min. Commuters using larger buses^25^ experienced a reduction from 76 to 58 min. Averaging those values, a commuter saw a reduction of 16 min in his/her commuting trip—half the magnitude of official reports (see Bel and Holst 2018, p.212). We translate these time savings into monetary terms by utilizing our estimates of value of commuting time for commuters taking bus and with a commute longer than 50 min—i.e. MXP 750 (USD 39.39) on a hourly basis, which is equivalent to USD 0.65 per minute. Thus, benefits from introduction of BRT system in Mexico City arising from reduction of commuting times can be valued at (2019) USD 10.50. As this number refers to one-way trips, the round-trip value is USD 21.10. Considering 20 two-round trips, monthly time savings are valued at USD 420.16 per commuter—which is USD 100 more than the average monthly total commuting costs in our sample.

Welfare estimates reported in this paper offer insights into potential benefits from affordable housing policies in a post-COVID-19 era. With over 90% of COVID-19 cases occurring in urban areas (United Nations 2020), it has become clear that housing design play a relevant role in building resilience of cities. Consequently, scholars and practitioners are rethinking affordable housing and its link to urban sustainability (Rogers and Power 2020). Lack of appropriate spaces to perform office work at home is a documented challenge faced by office workers in overcrowded Mexico City during the COVID-19 pandemic (Arellano García 2020; Mendoza Rojas 2020; Vazquez 2015). Thus it is not a stretch to entertain the idea that provision of an office at home will become a matter of affordable housing policy. In this context, our average estimates for teleworking with no commuting time can be interpreted as reflecting stated benefits from including an office space in house. Thus the average benefits from an office-at-home policy would be equivalent to (2019) MXP 5,475 or USD 300 on a monthly basis. Consistent with economic theory, this number is strikingly similar to MXP 5,400 which reflect total monthly commuting costs—assuming 20 two-way trips. That is, the value from providing an office at home is equivalent to the savings from not commuting.

Estimates reported in this paper also offer insights into benefits from labor regulations. For instance, a reform to the Mexican Labor Law took place in January 11, 2021, establishing that office workers are allowed to work remotely from home or a location chosen by him/her (STPS 2020). As part of this regulation, employers are in charge of buying and delivering office equipment needed by employees, and paying a percentage of employees’ internet and energy bills. The regulation also establishes that employers are not allowed to reduce salaries of employees working remotely.

When it comes to translating this labor regulation into practice, teleworking from near-home shared offices likely will be preferred by office workers and their employers over the option of home-based teleworking. As employees are allowed to work remotely from a location chosen by them, and given that home-based teleworking has faced the challenge that no appropriate space is available to perform office work at home (Arellano García 2020; Mendoza Rojas 2020; Vazquez 2015), it is reasonable to entertain the idea that office workers may choose to telework from an office space near their homes. At the same time, as employers are mandated to afford features of an office environment, it is also reasonable that employers will find that renting office spaces is more straightforward than acquiring equipment and accommodating specific needs that their employees may have at home—i.e. shared offices offer the possibility of economies of scale to employers, and potentially will decrease total renting costs. The idea that teleworking from near-home shared offices will be preferred over home-based teleworking is consistent with features of business models launched in Mexico amid COVID-19. As of November 2020, hotels and restaurants have developed affordable near-home shared office environments aiming to attract office workers unsatisfied with home-based teleworking (Forbes 2020; Valadez 2020).

In this context, we highlight that our discrete choice experiment allows for estimation of benefits of teleworking from a shared office across commuting time scenarios—i.e. we only need to subtract value of commuting time from WTP for teleworking. For instance, WTP for teleworking in a shared office located 30 min away from a worker’s home is estimated at USD 45.69 on a monthly basis. This number can be interpreted as the increase in welfare that office workers would experience if teleworking from an office environment that is located 30 min away home. This increase in workers’ welfare would originate in the change of labor regulations —an empirical evaluation of such benefits requires information about commuting time to teleworking premises chosen by workers in practice. This interpretation, indeed, assumes that renting costs are covered by employers—which is a reasonable assumption given that, by regulation, employers are mandated to provide an office environment to their employees.

## Conclusions and discussion

Based on analysis of a discrete choice experiment implemented on office workers in Mexico City prior to COVID-19, we report estimates of stated willingness to pay (WTP) for teleworking from a shared office—(2019) MXP 182.5 (USD 9.58) on a daily basis, if commuting time is zero. We also report estimates of value of commuting time—MXP 73.75 (USD 3.87). Together, estimates of WTP for teleworking and value of one hour of commuting time can inform estimates of WTP for teleworking in a shared office under different commuting time scenarios—Sect. 6.2 illustrates this point. A third parameter of interest in this paper is WTP for bike parking infrastructure MXP 280—USD 14.07 on a monthly basis.

As economic theory suggests, heterogeneity in both WTP for teleworking and value of commuting time is associated with income, commuting time, and commuting mode. A general finding is that wealthier respondents—with wealth reflected either through income or through use of private car—who spend less-than-the-median commuting time report higher WTP for teleworking and higher values of commuting time—Sect. 5.2 provides details about observed heterogeneity patterns. Further exploration of factors explaining heterogeneity in preferences is warranted. In particular, this paper has not explored spatially- and/or regionally-driven differences in WTP—e.g. keeping commuting time and/or distance fixed, there may be differences in WTP explained by whether office workers commute from the north o the south of CDMX.

As findings in this paper suggest, shared offices as premises for teleworking are attractive alternatives for office workers in Mexico City even before COVID-19. In this sense, estimates reported here can be thought as baseline stated benefits that may inform cost-benefit analysis of policies enabling teleworking and/or decreasing commuting time in Mexico City and, under reasonable transfer benefit assumptions, in megacities of emerging economies where these estimates are absent—Sect. 6.2 illustrates this point for Mexico City. The baseline feature of our estimates arises from the fact that data collection occurred three months before the initial date that COVID-19 pandemic officially reached Mexico.^26^

Preferences for teleworking have likely shifted during the pandemic. The direction of this shift is to be determined empirically but anecdotal evidence suggest that interest of office workers for teleworking has increased through the pandemic. For instance, as lockdown measures have weaken worldwide, office workers in Latin America—including Mexico—have reported a strong preference for teleworking alternatives as part of the *new normal* (Microsoft WorkLab 2021).

Exploring potential benefits from a widespread adoption of teleworking from shared offices is also relevant for developed countries because ongoing policies or projects—both private and public ones—can be informed with preferences for teleworking. For instance, co-working start-ups in USA are betting on the suburbs with the premise that the pandemic has spawned a new kind of worker who wants an office space closer home to avoid long commuting (Hong and Haag 2021). These start-ups’ bet seems backed up by the fact that, as lockdown restrictions are waning worldwide, most office workers in developed countries have preferred hybrid working arrangements—e.g. as of November 2021, around 40% of all American working hours are still spent at home (The Economist 2021). In Europe preferences for shared offices can be explored, for instance, as part of the research agenda that is documenting preferences of potential residents of Positive Energy Districts (PED)—residential communities that combine “built environment, sustainable production and consumption, and mobility to reduce energy use and greenhouse gas emission and to create added value and incentives for the consumer” (European Commission 2018). Mihailova et al. (2022) have recently documented that shared spaces play a smaller positive or even negative role in Swiss residents’ preferences for PED configurations but these preferences may change once the COVID-19 pandemic is over as shared spaces may become instrumental to incorporate hybrid work arrangements into the *new normal*.

Ongoing and future research by the authors of this paper aim to explore and inform the design of optimal networks of shared offices across Mexico City—these networks would optimize societal net benefits reflecting workers’ direct benefits and positive externalities to the entire society. The premise behind this exploration is that, if widely adopted in Mexico City, teleworking from a near-home shared office may reduce work-related trips and, in turn, alleviate congestion during peak hours. It may also reduce net energy consumption and corresponding carbon emissions when considering work and non-work travel, and home and office energy use.^27^ While recent literature suggests that these net energy savings may in reality be modest or non-existent (Hook et al. 2020; O’Brien and Aliabadi 2020), these conclusions are based on literature that focuses on home-based teleworking in cities of developed economies. A bigger picture is needed and it should include differences in energy efficiency in office spaces and dwellings in cities of emerging economies. This bigger picture would also consider benefits from teleworking in other domains such as value of saved commuting time and the potential for an increase in social capital. In this respect, promotion of teleworking from a near-home shared office can also be linked to arguments that, in 1960s, were used to label measures enabling teleworking as social policies that allow workers to spend more time with their families (Hook et al. 2020).

The COVID-19 pandemic has made clear that teleworking is an essential feature of resilient societies. Thus, from a big-picture point of view, this paper initiates a conversation about the possibility that a system of shared offices located within a reasonable distance from workers’ homes may represent an alternative for megacities to not only tackle congestion in peak hours and associated air pollution but to increase resilience in a post-COVID-19 world. Teleworking from a near-home shared office fits into the pursuing of a self-sufficient neighborhood or 15-minute cities, two concepts that have recently gained track among policy makers and politicians (e.g. C40CCLG 2020; Willsher 2020). These neighborhoods or cities would have offices, sports facilities, schools, medical centers, and shops within reasonable distance. Practitioners and policy makers in megacities may find this conversation of interest as these cities encompass sectors and regions with large potential benefits from teleworking—particularly, those located in emerging economies (Ansong and Boateng 2018; Bojovic et al. 2020).

## Appendix

See Tables 8, 9, 10, 11, 12, 13, 14, 15.

**Table 8:** Conditional and random parameter logit specifications on sub-samples defined based on income range

Attribute	Income below 15,000 MXP					Income above 15,000 MXP				
	Random parameters logit				Conditional Logit	Random parameters logit				Conditional Logit
	Price parameter is assumed					Price parameter is assumed				
	Normally-distributed		Fixed			Normally-distributed		Fixed		
	Random parameters are					Random parameters are				
	Uncorrelated	Correlated	Uncorrelated	Correlated		Uncorrelated	Correlated	Uncorrelated	Correlated	
*Mean parameters*										
1 if not status	3.142***	3.327***	2.048***	1.936***	1.238***	4.149***	4.572***	2.944***	2.901***	***1.588
Quo alternative [1]	(0.238)	(0.289)	(0.183)	(0.197)	(0.108)	(0.328)	(0.333)	(0.227)	(0.237)	(0.116)
Walking commuting	− 2.194***	− 2.479***	− 1.611***	− 1.625***	− 1.054***	− 3.818***	− 4.300***	− 3.154***	− 3.176***	− 1.790***
Time (hours) [2]	(0.211)	(0.245)	(0.173)	(0.190)	(0.124)	(0.320)	(0.331)	(0.256)	(0.265)	(0.136)
1 if infrastructure	0.724***	0.882***	0.634***	0.775***	0.456***	0.676***	0.685***	0.605***	0.659***	0.424***
For bike parking [3]	(0.0825)	(0.116)	(0.0723)	(0.0958)	(0.0597)	(0.108)	(0.135)	(0.0896)	(0.118)	(0.0636)
Reduction in paycheck	− 2.496***	− 2.513***	− 1.390***	− 1.362***	− 1.115***	− 2.511***	− 2.682***	− 1.493***	− 1.470***	− 1.047***
(thousand MXP) [4]	(0.170)	(0.170)	(0.0698)	(0.0725)	(0.0529)	(0.189)	(0.198)	(0.0864)	(0.0878)	(0.0563)
*Standard deviation of parameters*										
1 if not status	2.800***		2.639***			3.143***		2.858***		
quo alternative [1]	(0.212)		(0.175)			(0.314)		(0.207)		
Walking commuting	− 1.672***		1.233***			− 2.282***		2.250***		
Time (hours) [2]	(0.397)		(0.292)			(0.542)		(0.286)		
1 if infrastructure	− 0.261		− 0.236			1.020***		0.654**		
For bike parking [3]	(0.483)		(0.240)			(0.270)		(0.205)		
Reduction in paycheck	1.715***					1.716***				
(thousand MXP) [4]	(0.149)					(0.187)				
*Elements of lower-triangular matrix L, where covariance matrix of random coefficients is* $V = L^{'}L$										
L11		3.100***		2.752***			2.998***		2.906***	
		(0.341)		(0.234)			(0.372)		(0.267)	
L21		0.443		0.183			0.943**		0.190	
		(0.348)		(0.289)			(0.386)		(0.368)	
L31		0.0110		− 0.322**			0.0386		− 0.244	
		(0.179)		(0.126)			(0.194)		(0.150)	
L41		− 0.489**					− 0.172			
		(0.181)					(0.197)			
L22		− 0.305		1.251***			1.193***		2.166***	
		(0.260)		(0.327)			(0.312)		(0.309)	
L32		0.866***		− 0.0683			0.432**		0.185	
		(0.161)		(0.164)			(0.184)		(0.175)	
L42		− 1.206***					1.252***			
		(0.159)					(0.198)			
L33		0.477**		− 0.319			− 1.214***		0.573**	
		(0.174)		(0.216)			(0.193)		(0.234)	
L43		1.204***					0.809***			
		(0.182)					(0.202)			
L44		− 0.103					0.841***			
		(0.253)					(0.154)			
Observations	7440	7440	7440	7440	7440	6708	6708	6708	6708	6708
Respondents	620	620	620	620	620	559	559	559	559	559
Log-likelihood	− 1942.0	− 1928.5	− 2030.1	− 2026.5	− 2343.8	− 1707.5	− 1688.0	− 1762.6	− 1760.6	− 2110.6
AIC	3899.9	3885.1	4074.2	4073.0	4695.6	3430.9	3404.1	3539.3	3541.2	4229.2
BIC	3955.2	3981.9	4122.6	4142.2	4723.3	3485.4	3499.4	3587.0	3609.3	4256.5

Standard errors in parentheses

**p* < 0.10, ***p* < 0.05, ****p* < 0.001

**Table 9:** Conditional and random parameter logit specifications on sub-samples defined based on commuting time

Attribute	Commuting time below 50 min					Commuting time below 50 min				
	Random parameters logit				Conditional Logit	Random parameters logit				Conditional Logit
	Price parameter is assumed					Price parameter is assumed				
	Normally-distributed		Fixed			Normally-distributed		Fixed		
	Random parameters are					Random parameters are				
	Uncorrelated	Correlated	Uncorrelated	Correlated		Uncorrelated	Correlated	Uncorrelated	Correlated	
*Mean parameters*										
1 if not status	3.495***	3.450***	2.402***	2.289***	1.420***	3.996***	4.074***	2.471***	2.429***	1.416***
quo alternative [1]	(0.265)	(0.289)	(0.207)	(0.222)	(0.111)	(0.294)	(0.313)	(0.197)	(0.206)	(0.112)
Walking commuting	− 3.723***	− 3.692***	− 2.816***	− 2.825***	− 1.812***	− 2.318***	− 2.657***	− 1.627***	− 1.671***	− 1.015***
Time (hours) [2]	(0.300)	(0.298)	(0.223)	(0.237)	(0.133)	(0.234)	(0.258)	(0.185)	(0.195)	(0.128)
1 if infrastructure	0.745***	0.816***	0.632***	0.771***	0.425***	0.715***	0.807***	0.621***	0.685***	0.455***
For bike parking [3]	(0.0986)	(0.132)	(0.0811)	(0.113)	(0.0625)	(0.0999)	(0.117)	(0.0764)	(0.0944)	(0.0609)
Reduction in paycheck	− 2.403***	− 2.373***	− 1.401***	− 1.372***	− 1.031***	− 2.806***	− 2.803***	− 1.432***	− 1.405***	− 1.133***
(thousand MXP) [4]	(0.166)	(0.173)	(0.0781)	(0.0807)	(0.0548)	(0.192)	(0.200)	(0.0732)	(0.0752)	(0.0543)
*Standard deviation of parameters*										
1 if not status	3.124***		2.923***			2.625***		2.601***		
quo alternative [1]	(0.235)		(0.194)			(0.199)		(0.187)		
Walking commuting	1.990***		− 1.460***			2.036***		1.595***		
Time (hours) [2]	(0.395)		(0.320)			(0.318)		(0.258)		
1 if infrastructure	0.825***		0.438**			1.026***		− 0.321		
For bike parking [3]	(0.204)		(0.211)			(0.199)		(0.209)		
Reduction in paycheck	1.525***					1.984***				
(thousand MXP) [4]	(0.122)					(0.180)				
*Elements of lower-triangular matrix L, where covariance matrix of random coefficients is* $V=LL$										
L11		3.113***		3.038***			3.342***		2.583***	
		(0.360)		(0.269)			(0.397)		(0.248)	
L21		0.664*		0.121			0.263		0.292	
		(0.385)		(0.358)			(0.388)		(0.308)	
L31		− 0.0541		− 0.307**			0.0721		− 0.208	
		(0.204)		(0.140)			(0.192)		(0.137)	
L41		− 0.187					− 0.674***			
		(0.199)					(0.181)			
L22		0.231		− 1.457***			0.923**		1.516***	
		(0.332)		(0.403)			(0.456)		(0.293)	
L32		− 1.017***		− 0.0505			− 0.463*		− 0.00745	
		(0.168)		(0.174)			(0.251)		(0.158)	
L42		0.902***					0.344			
		(0.152)					(0.279)			
L33		− 0.477**		0.405*			− 1.127***		− 0.388**	
		(0.178)		(0.236)			(0.164)		(0.197)	
L43		− 1.297***					0.0336			
		(0.151)					(0.156)			
L44		− 0.114					1.864***			
		(0.215)					(0.164)			
Observations	7272	7272	7272	7272	7272	6876	6876	6876	6876	6876
Respondents	606	606	606	606	606	573	573	573	573	573
Log-likelihood	− 1840.7	− 1835.9	− 1906.4	− 1903.8	− 2263.0	− 1790.7	− 1782.0	− 1878.5	− 1876.8	− 2168.1
AIC	3697.4	3699.8	3826.7	3827.6	4533.9	3597.3	3592.1	3771.1	3773.7	4344.3
BIC	3752.5	3796.3	3874.9	3896.5	4561.5	3652.0	3687.8	3818.9	3842.0	4371.6

Standard errors in parentheses

**p* < 0.10, ***p* < 0.05, ****p* < 0.001

**Table 10:** Conditional and random parameter logit specifications on sub-samples defined based on total costs of commuting (monetary costs plus opportunity costs)

Attribute	Total cost below MXP 102.65					Total cost above MXP 102.65				
	Random parameters logit				Conditional Logit	Random parameters logit				Conditional Logit
	Price parameter is assumed					Price parameter is assumed				
	Normally-distributed		Fixed			Normally-distributed		Fixed		
	Random parameters are					Random parameters are				
	Uncorrelated	Correlated	Uncorrelated	Correlated		Uncorrelated	Correlated	Uncorrelated	Correlated	
*Mean parameters*										
1 if not status	3.314$$^{***}$$	3.309$$^{***}$$	2.063$$^{***}$$	1.913$$^{***}$$	1.196$$^{***}$$	4.259$$^{***}$$	4.226$$^{***}$$	2.848$$^{***}$$	2.832$$^{***}$$	1.622$$^{***}$$
quo alternative [1]	(0.271)	(0.282)	(0.197)	(0.215)	(0.110)	(0.301)	(0.307)	(0.213)	(0.216)	(0.113)
Walking commuting x	− 2.692$$^{***}$$	− 2.894$$^{***}$$	− 2.071$$^{***}$$	− 2.007$$^{***}$$	− 1.260$$^{***}$$	− 3.339$$^{***}$$	− 3.408$$^{***}$$	− 2.521$$^{***}$$	− 2.632$$^{***}$$	− 1.548$$^{***}$$
quo alternative [1]	(0.253)	(0.259)	(0.208)	(0.222)	(0.128)	(0.272)	(0.272)	(0.218)	(0.224)	(0.131)
1 if infrastructure	0.686$$^{***}$$	0.863$$^{***}$$	0.570$$^{***}$$	0.744$$^{***}$$	0.407$$^{***}$$	0.783$$^{***}$$	0.854$$^{***}$$	0.675$$^{***}$$	0.741$$^{***}$$	0.475$$^{***}$$
For bike parking [3]	(0.0975)	(0.137)	(0.0776)	(0.108)	(0.0617)	(0.104)	(0.124)	(0.0839)	(0.103)	(0.0615)
Reduction in paycheck	− 2.569$$^{***}$$	− 2.605$$^{***}$$	− 1.331$$^{***}$$	− 1.323$$^{***}$$	− 1.028$$^{***}$$	− 2.735$$^{***}$$	− 2.649$$^{***}$$	− 1.543$$^{***}$$	− 1.496$$^{***}$$	− 1.138$$^{***}$$
(thousand MXP) [4]	(0.177)	(0.188)	(0.0717)	(0.0753)	(0.0534)	(0.183)	(0.181)	(0.0832)	(0.0833)	(0.0557)
*Standard deviation of parameters*										
1 if not status	3.398$$^{***}$$		2.761$$^{***}$$			3.156$$^{***}$$		2.686$$^{***}$$		
quo alternative [1]	(0.282)		(0.190)			(0.256)		(0.198)		
Walking commuting	− 1.499$$^{**}$$		− 1.761$$^{***}$$			1.814$$^{***}$$		1.709$$^{***}$$		
Time (hours) [2]	(0.561)		(0.319)			(0.345)		(0.330)		
1 if infrastructure	− 0.869$$^{***}$$		0.230			− 1.163$$^{***}$$		0.653$$^{***}$$		
For bike parking [3]	(0.209)		(0.274)			(0.181)		(0.189)		
Reduction in paycheck	1.792$$^{***}$$					1.758$$^{***}$$				
(thousand MXP) [4]	(0.141)					(0.150)				
Elements of lower-triangular matrix L, where covariance matrix of random coefficients is $V=LL$										
L11		3.506$$^{***}$$		3.104$$^{***}$$			3.218$$^{***}$$		2.509$$^{***}$$	
		(0.366)		(0.264)			(0.382)		(0.256)	
L21		0.448		− 0.199			0.532		0.758$$^{**}$$	
		(0.368)		(0.339)			(0.362)		(0.384)	
L31		− 0.0199		− 0.385$$^{**}$$			− 0.270		− 0.256$$^{*}$$	
		(0.184)		(0.134)			(0.173)		(0.153)	
L41		− 0.210					− 0.141			
		(0.170)					(0.217)			
L22		− 0.617$$^{*}$$		1.842$$^{***}$$			0.868$$^{**}$$		1.193$$^{**}$$	
		(0.370)		(0.307)			(0.320)		(0.542)	
L32		0.229		0.0133			0.435$$^{**}$$		0.0620	
		(0.224)		(0.135)			(0.156)		(0.271)	
L42		0.117					1.332$$^{***}$$			
		(0.175)					(0.158)			
L33		− 1.211$$^{***}$$		0.152			− 1.032$$^{***}$$		0.750$$^{***}$$	
		(0.155)		(0.257)			(0.160)		(0.169)	
L43		0.657$$^{***}$$					0.747$$^{***}$$			
		(0.147)					(0.134)			
L44		1.759$$^{***}$$					0.625$$^{**}$$			
		(0.146)					(0.199)			
Observations	7056	7056	7056	7056	7056	7092	7092	7092	7092	7092
Respondents	588	588	588	588	588	591	591	591	591	591
Log-likelihood	− 1808.9	− 1798.2	− 1901.3	− 1897.5	− 2234.2	− 1825.0	− 1820.2	− 1904.4	− 1900.8	− 2224.7
AIC	3633.9	3624.4	3816.6	3815.0	4476.4	3666.0	3668.5	3822.7	3821.6	4457.4
BIC	3688.8	3720.4	3864.7	3883.6	4503.9	3721.0	3764.6	3870.8	3890.3	4484.9

Standard errors in parentheses

**p* < 0.10, ***p* < 0.05, ****p* < 0.001

**Table 11:** Random parameter logit (assuming price parameter is normally distributed and no correlation among random parameters) on subsamples based on intersection of categories by income and commuting time

Attribute	Income (MXP) and commuting time (minutes)			
	<15,000 MXP		>15,000 MXP	
	<50 mins	>50 mins	<50 mins	>50 mins
*Mean parameters*				
1 if not status quo alternative [1]	2.927$$^{***}$$	3.314$$^{***}$$	4.147$$^{***}$$	3.910$$^{***}$$
	(0.354)	(0.328)	(0.428)	(0.431)
Walking commuting time (hours) [2]	− 2.451$$^{***}$$	− 1.980$$^{***}$$	− 5.008$$^{***}$$	− 2.492$$^{***}$$
	(0.299)	(0.280)	(0.517)	(0.369)
1 if infrastructure for bike parking [3]	0.890$$^{***}$$	0.654$$^{***}$$	0.611$$^{***}$$	0.828$$^{***}$$
	(0.130)	(0.118)	(0.147)	(0.152)
Amount subtracted from monthly paycheck (thousand MXP) [4]	− 2.463$$^{***}$$	− 2.498$$^{***}$$	− 2.272$$^{***}$$	− 2.584$$^{***}$$
	(0.224)	(0.216)	(0.240)	(0.264)
*Standard deviation of parameters*				
1 if not status quo alternative [1]	2.997$$^{***}$$	3.154$$^{***}$$	3.230$$^{***}$$	2.810$$^{***}$$
	(0.311)	(0.330)	(0.335)	(0.457)
Walking commuting time (hours) [2]	0.360	1.807$$^{***}$$	− 2.942$$^{***}$$	− 1.753$$^{**}$$
	(0.594)	(0.471)	(0.559)	(0.635)
1 if infrastructure for bike parking [3]	− 0.635$$^{**}$$	0.584$$^{**}$$	0.917$$^{**}$$	0.981$$^{**}$$
	(0.235)	(0.244)	(0.401)	(0.340)
Amount subtracted from monthly paycheck (thousand MXP) [4]	1.483$$^{***}$$	1.755$$^{***}$$	− 1.575$$^{***}$$	1.508$$^{***}$$
	(0.183)	(0.193)	(0.211)	(0.223)
Observations	3456	3984	3816	2892
Observations	288	332	318	241
Log-likelihood	− 885.7	− 1045.5	− 936.4	− 747.0
AIC	1787.3	2107.0	1888.9	1509.9
BIC	1836.5	2157.3	1938.9	1557.7

Standard errors in parentheses

**p* < 0.10, ***p* < 0.05, ****p* < 0.001

**Table 12:** Conditional and random parameter logit specifications on sub-samples defined based on commuting mode (bus and private car)

Attribute	Bus					Private car				
	Random parameters logit				Conditional Logit	Random parameters logit				Conditional Logit
	Price parameter is assumed					Price parameter is assumed				
	Normally-distributed		fixed			Normally-distributed		Fixed		
	Random parameters are					Random parameters are				
	Uncorrelated	Correlated	Uncorrelated	Correlated		Uncorrelated	Correlated	Uncorrelated	Correlated	
Mean parameters										
1 if not status	3.485$$^{***}$$	3.335$$^{***}$$	2.297$$^{***}$$	2.244$$^{***}$$	1.296$$^{***}$$	3.917$$^{***}$$	4.093$$^{***}$$	2.717$$^{***}$$	2.466$$^{***}$$	1.530$$^{***}$$
quo alternative [1]	(0.300)	(0.315)	(0.218)	(0.238)	(0.122)	(0.389)	(0.445)	(0.302)	(0.313)	(0.153)
Walking commuting	− 2.242$$^{***}$$	− 2.296$$^{***}$$	− 1.654$$^{***}$$	− 1.685$$^{***}$$	− 1.002$$^{***}$$	− 3.826$$^{***}$$	− 4.021$$^{***}$$	− 2.952$$^{***}$$	− 3.063$$^{***}$$	− 1.823$$^{***}$$
time (hours) [2]	(0.240)	(0.274)	(0.211)	(0.225)	(0.139)	(0.390)	(0.470)	(0.328)	(0.347)	(0.183)
1 if infrastructure	0.720$$^{***}$$	0.871$$^{***}$$	0.599$$^{***}$$	0.697$$^{***}$$	0.425$$^{***}$$	0.560$$^{***}$$	0.685$$^{***}$$	0.532$$^{***}$$	0.754$$^{***}$$	0.352$$^{***}$$
for bike parking [3]	(0.0982)	(0.133)	(0.0832)	(0.107)	(0.0666)	(0.150)	(0.192)	(0.121)	(0.165)	(0.0855)
Reduction in paycheck	− 2.627$$^{***}$$	− 2.495$$^{***}$$	− 1.425$$^{***}$$	− 1.428$$^{***}$$	− 1.114$$^{***}$$	− 2.469$$^{***}$$	− 2.431$$^{***}$$	− 1.335$$^{***}$$	− 1.245$$^{***}$$	− 0.975$$^{***}$$
(thousand MXP) [4]	(0.199)	(0.190)	(0.0784)	(0.0873)	(0.0586)	(0.247)	(0.245)	(0.107)	(0.104)	(0.0744)
Standard deviation of parameters										
1 if not status	3.264$$^{***}$$		2.821$$^{***}$$			3.023$$^{***}$$		2.855$$^{***}$$		
quo alternative [1]	(0.268)		(0.221)			(0.320)		(0.282)		
Walking commuting	1.508$$^{***}$$		1.607$$^{***}$$			− 1.958$$^{***}$$		− 1.670$$^{***}$$		
time (hours) [2]	(0.452)		(0.336)			(0.439)		(0.492)		
1 if infrastructure	0.533$$^{**}$$		0.159			− 1.133$$^{***}$$		− 0.770$$^{***}$$		
for bike parking [3]	(0.251)		(0.231)			(0.287)		(0.231)		
Reduction in paycheck	1.813$$^{***}$$					1.822$$^{***}$$				
(thousand MXP) [4]	(0.180)					(0.211)				
Elements of lower-triangular matrix L, where covariance matrix of random coefficients is $V=LL$										
L11		3.425$$^{***}$$		3.010$$^{***}$$			2.267$$^{***}$$		2.712$$^{***}$$	
		(0.407)		(0.294)			(0.416)		(0.338)	
L21		0.625$$^{*}$$		− 0.0222			1.462$$^{**}$$		1.084$$^{**}$$	
		(0.374)		(0.369)			(0.505)		(0.434)	
L31		− 0.217		− 0.331$$^{**}$$			− 0.215		− 0.586$$^{**}$$	
		(0.187)		(0.139)			(0.265)		(0.259)	
L41		− 0.0911					1.144$$^{***}$$			
		(0.266)					(0.212)			
L22		0.820$$^{**}$$		1.693$$^{***}$$			− 1.478$$^{***}$$		− 0.563	
		(0.412)		(0.373)			(0.374)		(0.593)	
L32		0.823$$^{***}$$		0.197			− 0.275		− 0.529	
		(0.190)		(0.139)			(0.304)		(0.925)	
L42		− 0.814$$^{***}$$					1.111$$^{***}$$			
		(0.194)					(0.206)			
L33		− 0.240		0.0605			− 1.407$$^{***}$$		0.470	
		(0.255)		(0.219)			(0.278)		(1.110)	
L43		− 0.455$$^{**}$$					0.186			
		(0.175)					(0.218)			
L44		− 1.440$$^{***}$$					− 0.350			
Observations	5832	5832	5832	5832	5832	3696	3696	3696	3696	3696
Respondents	486	486	486	486	486	308	308	308	308	308
Log-likelihood	− 1491.3	− 1481.1	− 1563.7	− 1560.0	− 1837.2	− 956.2	− 947.4	− 992.0	− 987.6	− 1172.6
AIC	2998.5	2990.2	3141.5	3140.0	3682.5	1928.4	1922.8	1998.1	1995.2	2353.2
BIC	3051.9	3083.6	3188.2	3206.7	3709.2	1978.1	2009.8	2041.6	2057.4	2378.1

Standard errors in parentheses

**p* < 0.10, ***p* < 0.05, ****p* < 0.001

**Table 13:** Conditional and random parameter logit specifications on sub-samples defined based on commuting mode (taxi/uber and metro)

Attribute	Taxi/uber					Metro				
	Random parameters logit				Conditional Logit	Random parameters logit				Conditional Logit
	Price parameter is assumed					Price parameter is assumed				
	Normally-distributed		fixed			Normally-distributed		fixed		
	Random parameters are					Random parameters are				
	uncorrelated	correlated	uncorrelated	correlated		uncorrelated	correlated	uncorrelated	correlated	
Mean parameters										
1 if not status	3.829***	4.587***	2.830***	2.873***	1.511***	3.558***	3.615***	2.419***	2.387***	1.492***
quo alternative [1]	(0.920)	(1.174)	(0.729)	(0.793)	(0.381)	(0.356)	(0.370)	(0.272)	(0.273)	(0.169)
Walking commuting	− 3.171***	− 3.073***	− 2.276**	− 2.066**	− 1.413**	− 2.962***	− 3.115***	− 2.366***	− 2.485***	− 1.549***
Time (hours) [2]	(0.909)	(0.869)	(0.693)	(0.681)	(0.432)	(0.363)	(0.366)	(0.309)	(0.317)	(0.198)
1 if infrastructure	0.750**	0.492	0.736**	0.601*	0.524**	0.745***	0.790***	0.656***	0.715***	0.514***
For bike parking [3]	(0.339)	(0.397)	(0.311)	(0.331)	(0.204)	(0.143)	(0.168)	(0.117)	(0.144)	(0.0936)
Reduction in paycheck	− 2.924***	− 3.216***	− 1.765***	− 1.799***	− 1.194***	− 2.380***	− 2.425***	− 1.333***	− 1.297***	− 1.065***
(thousand MXP) [4]	(0.633)	(0.697)	(0.308)	(0.313)	(0.185)	(0.238)	(0.250)	(0.107)	(0.109)	(0.0828)
Standard deviation of parameters										
1 if not status	− 3.125***		2.860***			2.118***		2.118***		
quo alternative [1]	(0.791)		(0.616)			(0.274)		(0.236)		
Walking commuting	2.508**		1.000			2.183***		1.780***		
Time (hours) [2]	(0.957)		(1.358)			(0.461)		(0.397)		
1 if infrastructure	− 1.004		1.032*			− 0.898***		0.337		
For bike parking [3]	(0.914)		(0.541)			(0.265)		(0.294)		
Reduction in paycheck	1.712***					1.663***				
(thousand MXP) [4]	(0.462)					(0.206)				
Elements of lower-triangular matrix L, where covariance matrix of random coefficients is $V=LL$										
L11		3.084**		3.107***			2.562***		1.825***	
		(1.392)		(0.885)			(0.580)		(0.312)	
L21		− 0.0775		− 0.573			− 0.333		0.962	
		(1.325)		(0.918)			(0.669)		(0.596)	
L31		1.214		0.285			0.608*		− 0.0900	
		(0.740)		(0.461)			(0.319)		(0.251)	
L41		0.184					− 0.292			
		(0.540)					(0.230)			
L22		0.108		− 0.888			2.125***		− 1.009	
		(0.732)		(0.979)			(0.545)		(0.854)	
L32		− 0.581		− 0.343			− 0.228		0.438	
		(0.662)		(0.569)			(0.434)		(0.338)	
L42		1.186**					0.222			
		(0.550)					(0.314)			
L33		− 0.307		0.743			− 0.409		0.475	
		(0.673)		(0.577)			(0.288)		(0.295)	
L43		− 0.763					1.045***			
		(0.539)					(0.305)			
L44		0.916**					1.219***			
		(0.373)					(0.309)			
Observations	660	660	660	660	660	3072	3072	3072	3072	3072
Respondents	55	55	55	55	55	256	256	256	256	256
Log-likelihood	− 164.3	− 161.9	− 170.6	− 170.0	− 203.4	− 827.4	− 824.6	− 864.0	− 863.5	− 973.3
AIC	344.5	351.8	355.1	360.1	414.9	1670.8	1677.1	1741.9	1747.1	1954.5
BIC	380.5	414.7	386.6	405.0	432.8	1719.0	1761.5	1784.1	1807.4	1978.6

Standard errors in parentheses

**p* < 0.10, ***p* < 0.05, ****p* < 0.001

**Table 14:** Conditional and random parameter logit specifications on sub-samples based on commuting mode (motor- or bicycle, or walking)

Attribute	Random parameters logit				Conditional Logit
	Price parameter is assumed				
	Normally-distributed		Fixed		
	Random parameters are				
	Uncorrelated	Correlated	Uncorrelated	Correlated	
Mean parameters					
1 if not status quo alternative [1]	4.220***	3.851**	4.210***	3.453**	1.389***
	(1.130)	(1.441)	(1.133)	(1.182)	(0.351)
Walking commuting time (hours) [2]	− 5.793***	− 5.910***	− 5.754***	− 4.475***	− 1.998***
	(1.359)	(1.787)	(1.330)	(1.254)	(0.426)
1 if infrastructure for bike parking [3]	1.482**	2.481**	1.480**	1.706**	0.687***
	(0.501)	(1.226)	(0.499)	(0.681)	(0.199)
Amount subtracted from monthly paycheck	− 3.563***	− 3.898***	− 3.533***	− 3.581***	− 1.417***
(thousand MXP) [4]	(0.672)	(0.944)	(0.655)	(0.654)	(0.196)
Standard deviation of parameters					
1 if not status quo alternative [1]	5.118***		5.113***		
	(1.056)		(1.055)		
Walking commuting time (hours) [2]	3.389**		3.357**		
	(1.138)		(1.107)		
1 if infrastructure for bike parking [3]	2.567***		2.520***		
	(0.773)		(0.743)		
Amount subtracted from monthly paycheck	0.154				
(thousand MXP) [4]	(0.549)				
Elements of lower-triangular matrix L, where covariance matrix of random coefficients is $V = L^{'}L$					
L11		9.521**		5.954***	
		(3.030)		(1.416)	
L21		− 3.129		− 1.171	
		(2.045)		(1.322)	
L31		0.195		− 0.146	
		(0.949)		(0.684)	
L41		− 2.049			
		(1.261)			
L22		− 1.241		− 0.677	
		(1.498)		(1.076)	
L32		2.458**		0.958	
		(0.983)		(0.702)	
L42		− 1.040			
		(0.638)			
L33		− 2.972**		− 2.353**	
		(1.142)		(0.740)	
L43		− 0.311			
		(0.561)			
L44		− 0.358			
		(0.433)			
Observations	888	888	888	888	888
Respondents	74	74	74	74	74
Log-likelihood	− 182.2	− 177.3	− 182.2	− 180.2	− 251.8
AIC	380.4	382.6	378.5	380.3	511.6
BIC	418.7	449.7	412.0	428.2	530.8

Standard errors in parentheses

**p* < 0.10, ***p* < 0.05, ****p* < 0.001

**Table 15:** Random parameter logit (assuming price parameter is normally distributed and no correlation among random parameters) on subsamples based on intersection of categories by commuting mode and commuting time

Attribute	Commuting time (minutes)									
	< 50 mins					> 50 mins				
	Bus	Private car	Taxi/uber	Metro	Motor- or bicycle, or walking	Bus	Private car	Taxi/uber	Metro	Motor- or bicycle, or walking
Mean parameters										
1 if not status	2.752***	4.416***	4.555***	3.335***	4.043**	4.343**	3.073***	3.481***	3.154**	7.217*
quo alternative [1]	(0.394)	(0.494)	(0.586)	(0.535)	(1.347)	(1.612)	(0.473)	(0.491)	(1.081)	(4.143)
Walking commuting	− 2.488***	− 2.112***	− 5.027***	− 3.054***	− 4.409**	− 1.436	− 3.704***	− 2.132***	− 5.363***	− 5.445*
time (hours) [2]	(0.358)	(0.343)	(0.709)	(0.520)	(1.511)	(1.085)	(0.598)	(0.466)	(1.265)	(3.185)
1 if infrastructure	0.766***	0.717***	0.365*	0.891***	0.333	1.238**	0.856***	0.709***	1.985***	0.930
for bike parking [3]	(0.145)	(0.142)	(0.218)	(0.213)	(0.444)	(0.602)	(0.193)	(0.199)	(0.566)	(1.206)
Reduction in paycheck	− 2.427***	− 2.797***	− 2.462***	− 2.484***	− 3.080**	− 3.106***	− 1.938***	− 2.572***	− 3.388***	− 4.399*
(thousand MXP) [4]	(0.282)	(0.280)	(0.388)	(0.412)	(1.063)	(0.936)	(0.274)	(0.344)	(0.679)	(2.354)
Standard deviation of parameters										
1 if not status	3.430***	3.579***	− 2.837***	3.111***	2.895**	2.827**	2.489***	1.842***	5.552***	6.771
quo alternative [1]	(0.429)	(0.401)	(0.456)	(0.517)	(0.982)	(1.101)	(0.414)	(0.402)	(1.539)	(4.144)
Walking commuting	− 0.993*	− 1.295**	2.567***	1.746***	0.599	− 0.961	1.944**	2.382***	1.889**	− 1.292
time (hours) [2]	(0.583)	(0.430)	(0.602)	(0.495)	(3.101)	(1.171)	(0.865)	(0.559)	(0.881)	(2.015)
1 if infrastructure	− 0.396	0.728**	− 1.284***	− 1.002***	− 0.0702	1.872*	− 0.532	− 0.741	1.871**	− 2.726
for bike parking [3]	(0.335)	(0.293)	(0.346)	(0.295)	(0.890)	(1.000)	(0.536)	(0.485)	(0.700)	(1.962)
Reduction in paycheck	1.593***	1.997***	1.688***	1.557***	1.758**	1.629**	0.974***	1.904***	0.568	0.793
(thousand MXP) [4]	(0.218)	(0.238)	(0.280)	(0.383)	(0.695)	(0.652)	(0.245)	(0.305)	(0.579)	(1.051)
Observations	2628	3204	1968	1728	312	348	1596	1476	768	120
Respondents	219	267	164	144	26	29	133	123	64	10
Log-likelihood	− 666.3	− 814.1	− 498.4	− 445.8	− 72.74	− 83.72	− 419.9	− 401.4	− 153.3	− 26.10
AIC	1348.6	1644.2	1012.7	907.6	161.5	183.4	855.7	818.9	322.5	68.21
BIC	1395.6	1692.7	1057.4	951.2	191.4	214.3	898.7	861.2	359.7	90.51

Standard errors in parentheses

**p* < 0.10, ***p* < 0.05, ****p* < 0.001

## Footnotes

1 For instance, a trade-off between travel time and travel cost between a car and a bus.

2 For instance, keeping mode constant, a trade-off between time and cost originated in the possibility of taking different routes, lanes, or destinations.

3 This trade-off has also been studied in the labor market—workers who are willing to commute a greater distance likely request a higher wage. Studies belonging to this literature are mostly based on revealed preferences data—e.g. Isacsson et al. (2013), French et al. (2020), Fu and Viard (2019), Russo et al. (2012), and Van Ommeren and Fosgerau (2009).

4 Rouwendal and Meijer (2001) represents an earlier DCE application exploring the value of commuting time through the trade-off among wage, commuting time and housing attributes.

5 Pioneer derivations of the log sum formula were independently developed by Ben-Akiva (1973) and McFadden (1973).

6 Further details can be found in Haab and McConnell (2002)

7 A third limitation is that a CL is not fitted to capture correlation over time (Train 2009).

8 To increase the chances that approached potential respondents agree to answer our survey, enumerators were instructed to approach them when upon arrival to the location of contact—i.e. enumerators waited to see office workers leaving their office buildings to take a break.

9 Graizbord (2015) has identified producer services and wholesale trade as the two sectors with the most potential to promote teleworking from home in Mexico City. Based on a survey answered by 72 teleworkers and their supervisors in Mexico City in 2011, he has estimated that greenhouse gas emissions of teleworkers decrease in 9% once a rebound effect is taken into consideration.

10 This term has been presented in English in the original text as it reflect the way shared offices are called among office workers and companies in Mexico City.

11 The original text reads as follows: *Las siguientes preguntas buscan conocer sus preferencias y percepciones en caso de que se le ofreciera la posibilidad de trabajar cerca de su casa, en un edificio de oficinas compartidas. Estas oficinas también son conocidas como *coworkings* y son oficinas adaptadas para que varios usuarios puedan trabajar en un mismo espacio en diferentes momentos. Las personas interesadas en trabajar en oficinas compartidas tendrán acceso a todos los servicios, infraestructura y comodidades necesarias para llevar a cabo su trabajo.*

12 The original text reads as follows: *Imagine que su empleador o empleadora le permite trabajar 2 días a la semana desde una oficina compartida. Usted pagaría una membresía mensual, en la forma de un descuento de nónima, para acceder a tales oficinas. En el momento que ya no le sea conveniente, usted tendrá la opción de ir a su trabajo todos los días de la semana.*

13 We have used the Stata command *dcreate*, documented by Risa-Hole (2015). In contrast to commands in other software, *dcreate* does not report a measure of efficiency with respect to a full factorial design (e.g. SAS). Instead, it reports absolute D-efficiency measures which implies that a “design with a higher D-efficiency is more efficient than a design for the same experiment with a lower D-efficiency, but the number themselves don’t mean much [...]” (Risa-Hole 2016). In our case, our final design has a D-efficiency of 1.73 in comparison to 0.63 of our initial design.

14 We use a exchange rate of 19.04 MXP/USD, which was in place as of December 12th, 2019.

15 We have collected responses from 1,200 office workers. We have dropped 21 observations due to missing values in one or more variables reported in Table 2.

16 To the best of our knowledge, no previous study has explicitly documented commuting time and costs of office workers in CDMX. Thus, for comparison purposes we use population average reported by an official source—i.e. (INEGI 2018).

17 Given that preferences for commuting time are negative and preferences for teleworking are positive, the positive correlation indicates that *the less a respondent dislikes commuting time* (the less negative the coefficient or the closer to zero), *the more he/she likes a teleworking alternative*. However, we would expect that the less he/she dislikes commuting time, the more willing he/she would be to commute, and *the less he/she likes a teleworking alternative*.

18 This strategy has proved useful in a wide range of contexts. The seminal application refers to estimation of economic benefits from rural landscape improvements in Ireland (Campbell 2007). Abildtrup et al. (2013) use this strategy to incorporate individual-specific spatial factors associated with heterogeneity in preferences for recreational use of forests in Lorraine, France. Yao et al. (2014) account for attitudes towards a planted forests program in New Zealand when estimating value of diversity enhancement. Zemo et al. (2019) explore individual- and household-specific determinants of WTP to reduce power outages in Ethiopia. Ghosh et al. (2021) explore determinants of farmers’ WTP for a subsidize insurance program in India.

19 Multi-mode commuters have assigned the commuting mode that they spent most of their commuting time.

20 Findings from OLS and SUR specifications on WTP for bike parking infrastructure and value of commuting time are available upon request from the corresponding author.

21 We have also explored a number of specifications on sub-samples defined based on gender, age, marital status, job sector, commuting routines, type of office space at employer’s premise, job-related meetings per month, and social gatherings. In general, specifications on sub-samples confirm findings reported in Table 5—i.e. WTP remains fairly homogeneous across categorizations based on those variables. Findings from these specifications are available upon request from the corresponding author.

22 Personal communication with administrative personnel of an NGO leasing shared space from WeWork.

23 Numbers are reported in Euros in original study. We have used an exchange rate of 1USD/1.11 Euros, which was in place in December 12th, 2019.

24 Vehicles with a capacity for 35 passengers (Wöhrnschimmel et al. 2008).

25 Vehicles with a capacity for 88 passengers(Wöhrnschimmel et al. 2008).

26 According to Wikipedia, the first case in Mexico was officially reported in February 28, 2020 (https://en.wikipedia.org/wiki/COVID-19_pandemic_in_Mexico).

27 As teleworking can be seen as a measure potentially impacting air quality, researchers and policy makers interested in intersecting both issues will likely face a number of documented challenges (and opportunities) when aiming to informed policy making with scientific evidence—as reported by Muñoz-Pizza et al. (2022) for seven cities in Mexico, including Mexico City.
